# Mapping Embryonic Mouse Lung Development Using Enhanced Spatial Transcriptomics

**DOI:** 10.1002/advs.77678

**Published:** 2026-09-27

**Authors:** Pengfei Zhang, Benjamin K. Law, Niles Huang, Katharine Goodwin, Celeste M. Nelson, Michelle M. Chan

**Affiliations:** ^1^ Department of Chemical and Biological Engineering Princeton University Princeton New Jersey USA; ^2^ Lewis‐Sigler Institute for Integrative Genomics Princeton University Princeton New Jersey USA; ^3^ Omenn‐Darling Bioengineering Institute Princeton University Princeton New Jersey USA; ^4^ Department of Molecular Biology Princeton University Princeton New Jersey USA

**Keywords:** cell‐cell interaction, morphodynamics, morphogenesis, pulmonary mesenchyme

## Abstract

The mature mammalian lung contains ∼50 different cell types that are located in precise positions along and around the airway epithelial tree. Here, we apply spatial transcriptomics to early embryonic mouse lungs to investigate how these spatial patterns are progressively established during development. Specifically, we optimized a microfluidics‐enabled, deterministic barcoding‐based sequencing (DBiT‐seq) workflow to achieve sensitive spatial mapping of tissue sections, resulting in up to a twofold increase in average transcript recovery compared with previous implementations. Spatial mapping of epithelial populations revealed that *Sox9* ^+^ epithelial progenitors are located throughout the epithelial tree at early stages before becoming confined to the distal ends. Moreover, we describe a previously unrecognized mesenchymal cluster that exhibits consistent spatial position, expresses high levels of extracellular matrix proteins, and appears to promote embryonic lung innervation. These findings provide new insights into the spatial organization and cellular dynamics underlying early lung development, and demonstrate the power of spatial transcriptomics to uncover hidden patterns and populations of cells.

## Graphical abstract

The mammalian lung contains ∼50 cell types arranged along the airway epithelium. Here, deterministic barcoding sequencing (DBiT‐seq) is optimized to nearly double transcript recovery, enabling highly sensitive spatial mapping of the embryonic mouse lung. Analysis reveals proximally located “distal” progenitors and a spatially restricted mesenchymal population linked to early lung innervation.

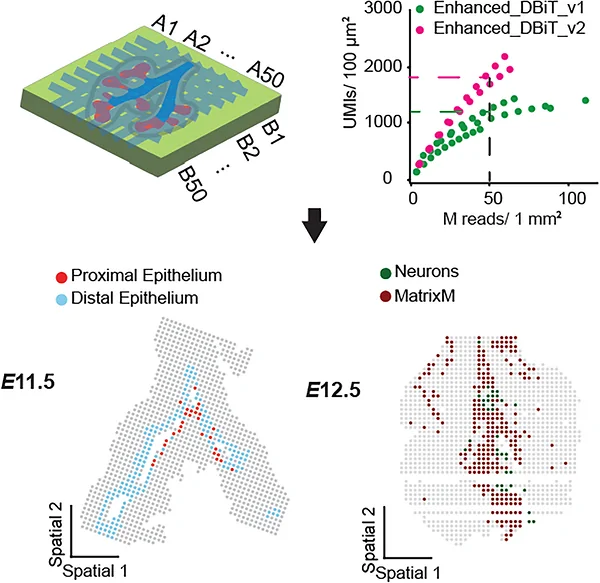

## 1 Introduction

The spatial architecture of the developing lung plays a key role in its morphogenesis. Initiated at embryonic day (*E*) 9.5, the mouse lung buds from the ventral foregut endoderm and then recursively branches to form a tree‐shaped structure [1, 2]. As the epithelium branches, the surrounding mesenchyme differentiates into diverse lineages that are localized to distinct regions throughout the lung. These tissues include mesothelium enveloping the outer surface of the lung, cartilage strengthening the trachea, smooth muscle wrapping around conducting airways, and pericytes supporting capillary endothelium. Collectively, the mesenchymal populations provide inductive signals that help specify proximodistal epithelial patterning, establishing *Sox2* ^+^ epithelium in the proximal airways and *Sox9* ^+^ epithelial progenitors at the distal tips [1, 2, 3].

An outstanding question in the field is the precise location of multipotent epithelial progenitors and how their location shifts during the establishment of the proximodistal axis in the developing lung epithelium [4]. Lineage‐tracing studies suggest that multipotent *Id2* ^+^ epithelial cells, located at the distal tips of the tree, contribute to both the proximal conducting airways and distal alveoli depending on the developmental stage [5]. Subsequent work showed that two nested waves–marked by the expression of SOX2 and SOX9 – demarcate a compartment boundary between proximal conducting airways and distal gas‐exchange epithelium [4]. However, the precise spatiotemporal distribution of multipotent progenitors, especially at early embryonic stages, remains largely undefined beyond insights from immunostaining or in situ hybridization of a limited set of targets [4, 5, 6, 7].

In parallel, a major challenge in studying the lung mesenchyme has been to classify this highly heterogeneous tissue into different subtypes. Clonal labeling experiments found that smooth‐muscle progenitors are spatially restricted to the mesenchyme positioned ahead of the branching epithelium, underscoring the importance of anatomical location in subtype specification [8]. Consistently, recent studies identified anatomically distinct mesenchymal subtypes that serve different roles in response to tissue injury and alveolar epithelial cell proliferation [9, 10]. Collectively, these findings highlight that spatial context is a key determinant of mesenchymal identity and function. Incorporating anatomical location into classification frameworks may therefore help to define mesenchymal subtypes and elucidate their roles in lung development.

Spatial transcriptomics offers the opportunity to complement single‐cell analysis and provide spatial context to the different cell types within a developing tissue, even those that are difficult to isolate into single cells for single‐cell RNA sequencing (scRNA‐seq), such as the epithelium [11, 12]. Consistently, efforts have recently been made to obtain comprehensive spatiotemporal maps of the embryonic lung [13, 14, 15, 16]. Unfortunately, current whole‐transcriptomic spatial‐mapping approaches suffer from limited resolution and/or sensitivity, hindering spatial mapping at the single‐cell level with high transcript detection sensitivity. Emerging technologies, such as Visium HD [17], Slide‐seqV2 [18], Seq‐Scope [19], Stereo‐seq [20], and OpenST [21], achieve single‐cell resolution but the detection sensitivity remains low due to inefficient mRNA diffusion and capture [21, 22]. In contrast, microfluidics‐based approaches, such as deterministic barcoding‐based sequencing (DBiT‐seq), enhance detection sensitivity by delivering spatial DNA barcodes directly into the tissue, bypassing barriers to mRNA diffusion [23] while achieving close‐to‐single‐cell resolution. Nevertheless, the transcript recovery for microfluidics‐based approaches remains low compared to scRNA‐seq [21, 22].

Here, we improved the detection sensitivity of spatial transcriptomics by optimizing the DBiT‐seq workflow [23]. Specifically, we optimized the tissue fixation conditions to better preserve mRNA integrity, incorporated a randomly primed second‐strand synthesis step to overcome the inefficiency in template switching, and relocated the reverse transcription (RT) from microchannels to a bulk reservoir to improve in situ RT efficiency. In total, these changes resulted in up to a twofold increase in transcript recovery. To highlight the benefits of this optimized workflow, we generated spatial datasets for tissue sections of the mouse lung at early embryonic stages. Combining our spatial transcriptomic data with reference scRNA‐seq allowed us to spatially map known cell types and uncover previously undescribed cell populations and tissue‐tissue interactions. We confirmed that proximal and distal epithelial cells exhibit distinct patterns of gene expression, but our spatial mapping unexpectedly revealed that ‘distal’ epithelial progenitor cells are located in proximal regions of the lung at early stages of development. Moreover, we identified a subset of mesenchymal cells enriched for extracellular matrix (ECM) markers that exhibited consistent spatial location patterns, which we designate as a novel “Matrix Mesenchyme” cell type. The Matrix Mesenchyme is located near the trachea and medial boundaries of the lung lobes and interacts with neurons to promote innervation of the developing organ [24, 25]. Together, these findings establish our optimized workflow and datasets as valuable resources for the community, offering new insights into the spatial organization and cellular dynamics of early lung development.

## 2 Results

### 2.1 An enhanced Spatial Transcriptomics Workflow

**FIGURE 1:**
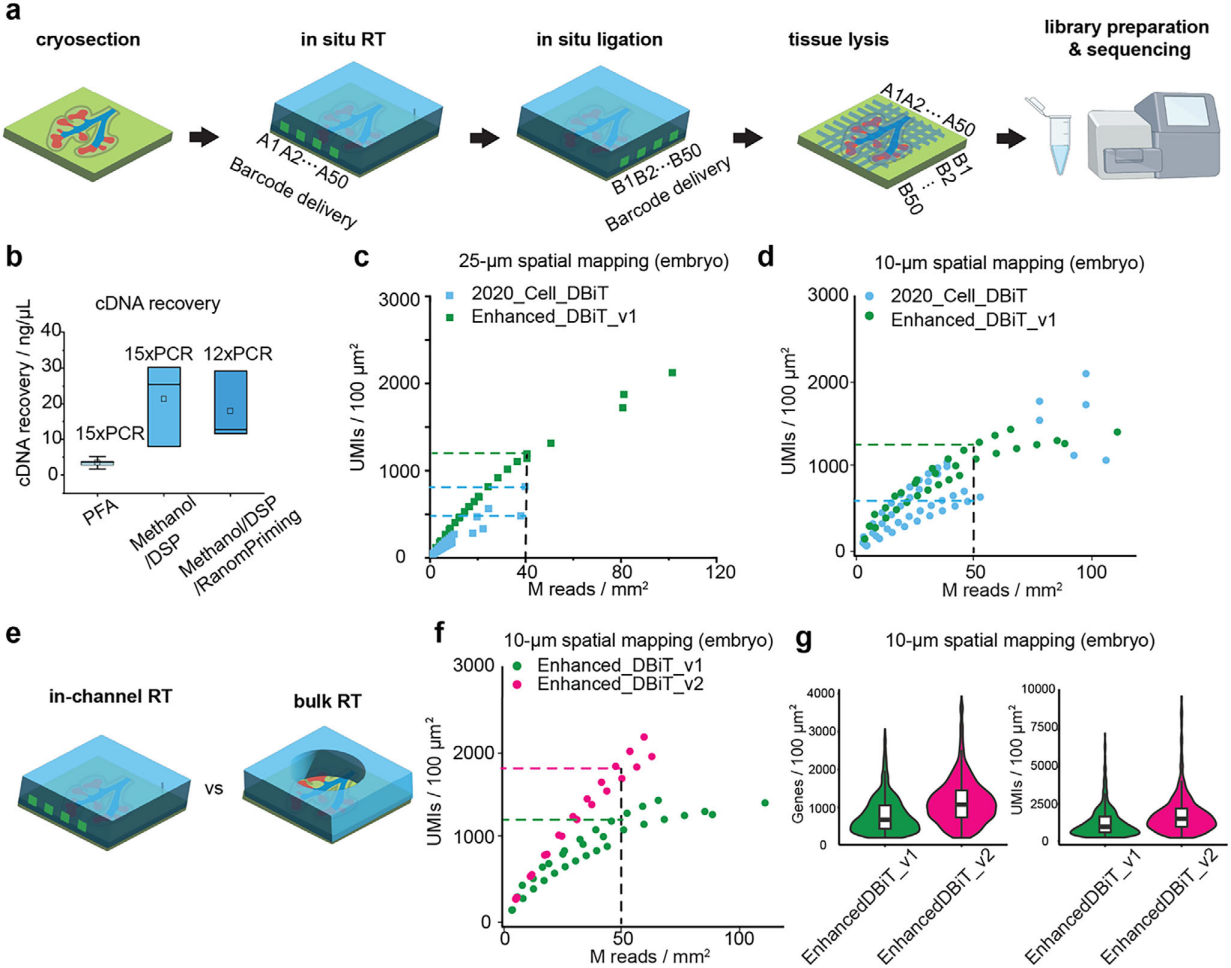
An enhanced spatial transcriptomic workflow. (a) Schematic overview of workflow for spatial transcriptomic mapping using microfluidics. (b) Graph of cDNA recovery as a function of fixation, with or without an additional random‐priming‐assisted, second‐strand synthesis step. (c) Saturation curve of 25‐µm tests using Enhanced_DBiT_v1 protocol (green) vs original DBiT‐seq protocol (blue) when testing whole embryo sections. (d) Saturation curve of 10‐µm spatial mapping of embryos using Enhanced_DBiT_v1 protocol (green) vs original DBiT‐seq protocol (blue). (e) Schematic showing comparison between two reverse transcription conditions: in channel vs bulk. (f) Saturation curves of 10‐µm spatial mapping of embryos using Enhanced_DBiT_v2 protocol (pink) vs Enhanced_DBiT_v1 protocol (green). (g) Violin plots of recovered UMIs/10‐µm pixel and genes/10‐µm pixel for Enhanced_DBiT_v1 vs Enhanced_DBiT_v2.

To investigate the spatial dynamics of the embryonic mouse lung, we optimized a microfluidics‐based DBiT‐seq workflow to enable unbiased, high‐resolution spatial mapping of the transcriptome (Figure 1 and Figures S1–S3). DBiT‐seq uses microfluidic chips to deliver oligonucleotides into tissue sections for spatial barcoding at a resolution that corresponds to the width of the channels on the chip (10–50 µm). Building upon a published DBiT‐seq workflow, we performed two rounds of optimization [23].

In the first round of optimization (Enhanced_DBiT_v1), we improved capture efficiency by changing tissue fixation conditions and incorporating a randomly primed second‐strand synthesis step (Figure 1a–d and Figures S1,S2) [18, 26, 27]. We found that adding diethyl pyrocarbonate (DEPC) in the fixation buffer increases cDNA yield, likely through inhibition of endogenous RNase activity [27]. We also observed that fixing with methanol/ dithiobis(succinimidyl propionate) (DSP) yields less‐fragmented cDNA compared to 4% paraformaldehyde (PFA). In addition, incorporating a random primed second‐strand synthesis step increases the cDNA recovery by mitigating the known inefficiency of template switching (Figure 1b and Figure S1). This optimized DBiT‐seq protocol resulted in a 55%–150% increase in unique molecular identifier (UMI) detection per pixel when tested on sections of whole embryos using 25‐µm‐resolution chips, after standardizing for pixel size and sequencing depth (Figure 1c). The Enhanced_DBiT_v1 workflow also recovers more UMIs (∼1000–1250 UMI/100 µm^2^) than the original DBiT‐seq protocol (∼600–1200 UMI/100 µm^2^) and the recently established Open‐ST (800 UMI/100 µm^2^) when tested on sections of whole embryos using 10‐µm‐resolution chips (Figure 1d) [21].

In the second round of optimization (Enhanced_DBiT_v2), we focused on improving the in situ reverse transcription (RT) step, which is known to be inefficient [28, 29, 30]. Performing RT within microchannels introduces an additional challenge due to nonspecific adsorption of reverse transcriptase to the polydimethylsiloxane (PDMS) surface of the chip. To address the issue, we decreased the PDMS surface area during the RT step by replacing the 10‐µm channels with a 5‐mm reservoir and modified the design of the barcoding assay (Figure 1e and Figure S3) [31]. This change further improved UMI and gene recovery, consistent with previous observations but achieved here at 10‐µm resolution (Figure 1f,g) [32]. As a result, the Enhanced_DBiT_v2 workflow recovered ∼1800 UMI/100 µm^2^ at 10‐µm resolution with a sequencing depth of 50 M reads/mm^2^. This UMI recovery is 0.5 to 2‐fold higher on average than that previously achieved using the original DBiT‐seq protocol (Figure 1d,f) and, our saturation analysis using Spacemake showed that Enhanced_DBiT has better detection sensitivity compared to Slide‐seqv2, Slide‐tags, and Visium, though it saturates faster than Slide‐tags (Figure S3b) [18, 22, 33]. We also note that difference in tissue type and sample preparation may contribute to the observed difference in sensitivity. Altogether, these improvements to tissue fixation and assay design enhance the detection sensitivity of microfluidic‐enabled spatial transcriptomics.

### 2.2 Using the Optimized Spatial Transcriptomics Workflow to Profile Early Development of the Embryonic Lung

**FIGURE 2:**
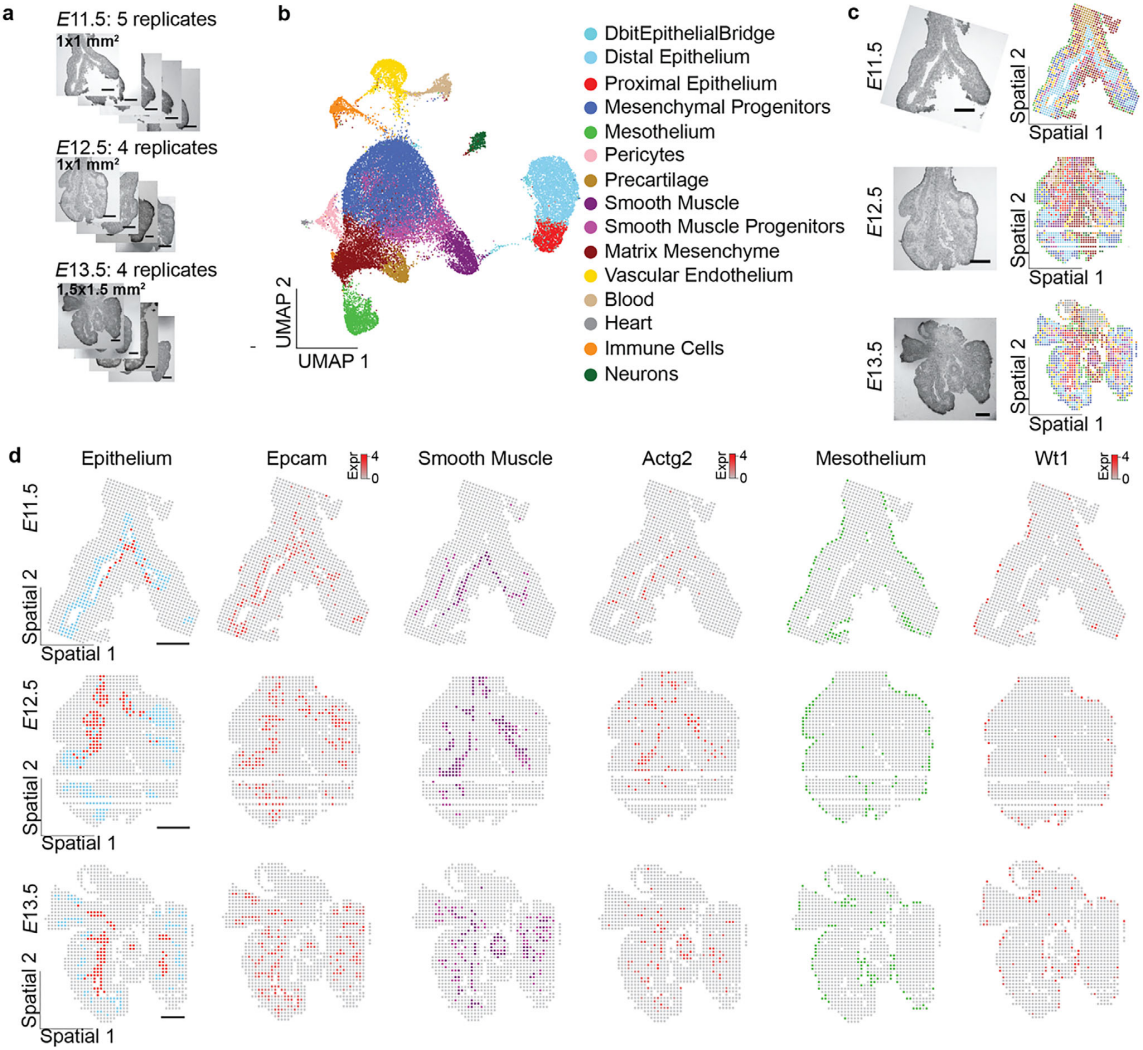
Spatial mapping of cell types in the embryonic mouse lung. (a) Brightfield images of replicates of embryonic lung sections. Scale bars: 200 µm. (b) UMAP showing cell‐type clustering for the embryonic mouse lung. (c) Brightfield images of representative sections and color‐coded maps of all cell types at each stage of development. Scale bars: 200 µm. (d) Color‐coded maps of epithelium, smooth muscle, and mesothelium, as well as their top gene markers at each stage. Scale bars: 200 µm.

We then evaluated the optimized workflow by spatially mapping the developing mouse lung. We focused on coronal sections of embryonic lungs acquired from *E*11.5–*E*13.5, when the epithelial tree is established (Figure 2a and Figure S4a). Specifically, we obtained 13 total replicates: 5 at *E*11.5 and 4 each at *E*12.5 and *E*13.5. The *E*11.5 and *E*12.5 replicates were analyzed at 10‐µm resolution, while the *E*13.5 replicates were analyzed at 15‐µm resolution to ensure we covered the entire tissue section. In total, we recovered 15 037 high‐quality pixels with a median value of 1 943 UMIs and 1 251 genes per replicate, consistent with tests using whole embryos (Figure 2b and Figure S5a,b). We observed a slightly higher recovery (2,221 UMIs and 1,480 genes per pixel, respectively) at *E*13.5, likely due to the larger pixel size.

To annotate cell identities, we first integrated our spatial transcriptomics data with published scRNA‐seq datasets and clustered the integrated data using the Louvain algorithm (Methods) [34, 35, 36, 37, 38]. Clusters, which contain both single cells and spatial pixels, were then annotated according to canonical marker genes, identifying major cell types such as Proximal and Distal Epithelium, Smooth Muscle, Mesothelium, and Mesenchymal Progenitors (Figure 2b and Figure S5c). We also identified a Heart cell cluster enriched for canonical cardiomyocyte markers, including *Actc1*, *Tnnt2*, *Tnni1*, and *Myl7*, and a Blood cell cluster marked by high expression of *Hba‐x*, *Hbb‐y*. To assess potential batch effects, we visualized the UMAP embedding split by sample and by developmental time point (Figure S6). Cell‐type composition is consistently represented across both time points and samples, with no evidence of systematic bias, indicating that batch effects do not substantially confound the downstream analysis. The proportions of annotated cell types are consistent within replicates of the scRNA‐seq and spatial transcriptomic data, respectively. However, we noticed that our spatial transcriptomics dataset captured a higher proportion of epithelial cells than the scRNA‐seq dataset (Figure S4c). This difference might be attributed to the fact that the latter approach requires dissociation of the organ into single cells, which has been shown to be challenging for epithelial tissues, including that of the embryonic lung [11, 12].

Mapping of the annotated cell types onto the tissue sections showed the expected locations, with an *Epcam* ^+^ epithelial tube surrounded by *Actg2* ^+^ smooth muscle cells and a peripheral layer of *Wt1* ^+^ mesothelium (Figure 2c,d), consistent across replicates and timepoints (Figure S7a–c). We also mapped the Heart and Blood cell clusters onto the tissue sections (Figure S8). The Heart cell cluster was found in only three replicates and localized near the carina of the lung, consistent with its likely origin as dissection carryover. The Blood cell cluster, meanwhile, localized adjacent to vascular endothelium, as expected for erythrocytes within the vasculature.

To determine whether the optimized protocol is close to resolving single cells per pixel, we carried out conditional autoregressive‐based deconvolution (CARD) [39]. This analysis generated similar spatial maps of the major cell types of the embryonic mouse lung (Figures S9,S10). However, we also found a portion of pixels that presented as a mixture of different cell types. For example, 21.5% of pixels annotated as epithelium were predicted to contain <50% epithelial cells. Across all cell types, 55.1% spatial pixels have a maximum predicted cell type proportion above 0.5, and different cell types showed a distinct degree of mixing, likely due to differences in transcriptomic similarity among cell types and the complexity of their local tissue microenvironment (Figure S10). The observed degree of mixed cell‐type signals is consistent with the challenge of guaranteeing single‐cell capture for spatial transcriptomics mapping [40]. Nevertheless, our optimized DBiT‐seq workflow enables high‐resolution (10‐µm) mapping of the major cell types of the early embryonic mouse lung with high sensitivity. To avoid any confounding issues from the possibility of cellular mixtures in spatial data, all differential gene‐expression analyses in the following sections were conducted using scRNA‐seq data, which is highlighted where it applies.

### 2.3 Spatiotemporal Mapping of the Epithelium Reveals Unexpected Pattern of *Sox9* ^+^ Progenitors

**FIGURE 3:**
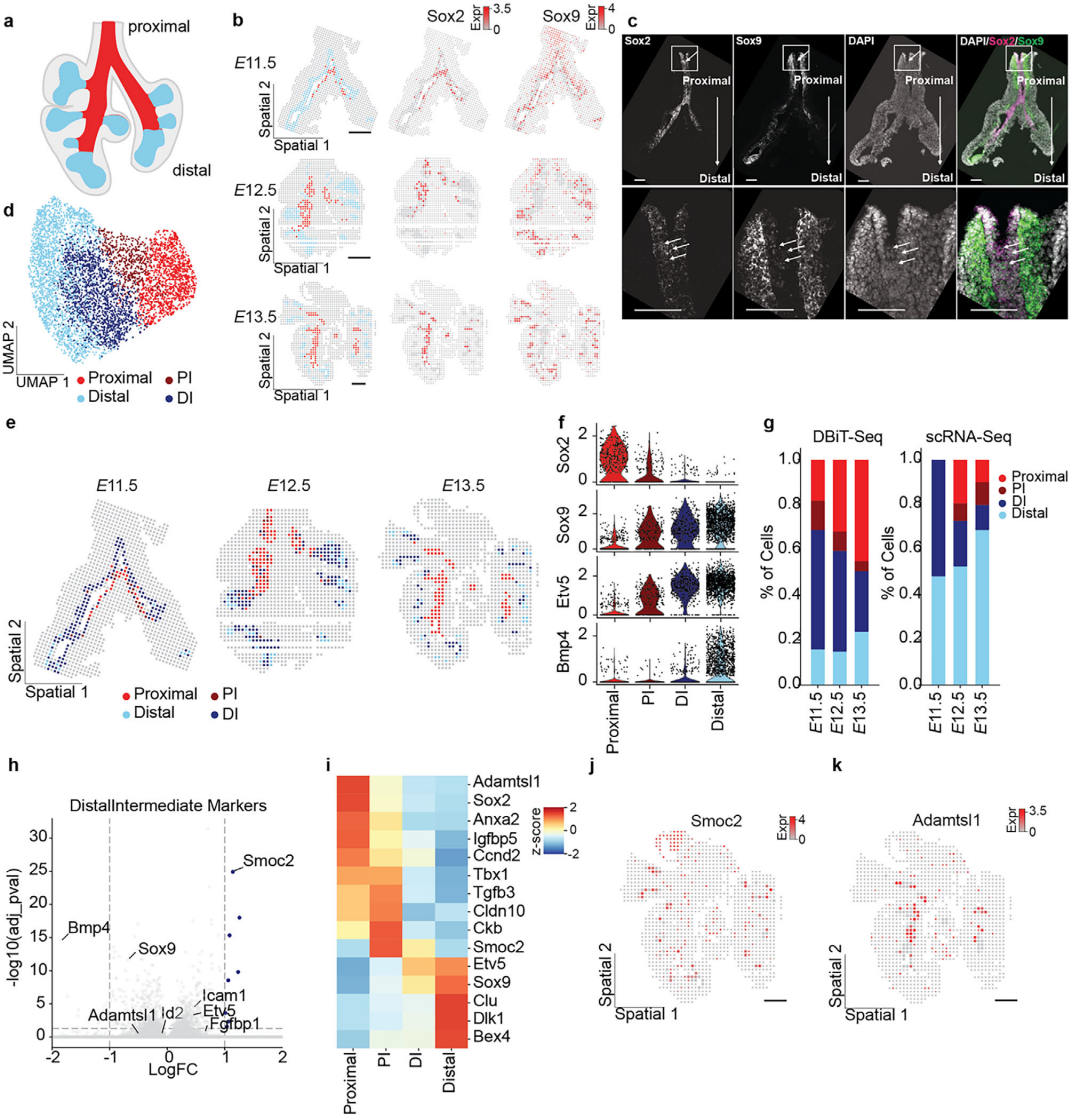
Spatiotemporal mapping of the epithelium reveals an unexpected pattern of multipotent progenitors. (a) Schematic of current conceptual model of lung epithelial development. (b) Color‐coded maps of proximal and distal epithelium and their top gene markers at each stage, including *Sox2* and *Sox9*. Scale bars: 200 µm. For ease of visualization, epithelial cells are highlighted with larger, darker pixels; spatial resolution is unchanged. PI: Proximal Intermediate; DI: Distal Intermediate. (c) Fluorescence images of hybridization analysis for DAPI, *Sox2*, and *Sox9* at each stage. Scale bars: 100 µm. The raw images were rotated and cropped to orient the trachea upward, and the empty space was filled with a rectangular black box. (d) UMAP of epithelial subclustering reveals 4 different subtypes. (e) Color‐coded maps of epithelial subclusters from (d). (f) Violin plots of *Sox2*, *Sox9*, *Etv5*, and *Bmp4* in scRNA‐seq data. (g) Bar plots of epithelial subcluster compositions for DBiT‐seq and scRNA‐seq. (h) Volcano plot of differential gene‐expression analysis between Distal Intermediate (DI) and the other epithelial cell subtypes using scRNA‐seq data. (i) Heatmap of the differentially expressed genes. (j,k) Color‐coded maps of *Smoc2* and *Adamtsl1* at *E*13.5. For ease of visualization, PI/DI and Proximal epithelial cells are highlighted with larger, darker pixels; spatial resolution is unchanged. Scale bars,:: 200 µm.

We took advantage of this dataset to investigate the differentiation trajectories and spatiotemporal distributions of the major tissues in the embryonic mouse lung. We first focused on the epithelium. According to previous lineage‐tracing studies, the lung epithelium is initially patterned along its proximodistal axis, with the distal *Sox9* ^+^ *Id2* ^+^ epithelial cells serving as the progenitors for different mature epithelial cell types (Figure 3a) [1, 2, 41]. While these *Sox9^+^Id2* ^+^ epithelial progenitors have been shown to localize to the distal regions of the epithelial tree from *E*11.5, it is not clear if they are spatially restricted or when that restriction may occur [5]. As a result, the precise spatiotemporal distribution of these progenitors, especially at early embryonic stages, remains largely undefined [4, 5, 6, 7].

Our spatial mapping provides a comprehensive delineation of the spatiotemporal pattern within the epithelium. Specifically, we identified two distinct epithelial clusters that express high levels of the proximal and distal epithelial markers, *Sox2* and *Sox9*, respectively (Figure 3b). However, the spatial mapping of these clusters reveals that the *Sox9* ^+^ ‘distal’ epithelial cells, annotated using additional markers including *Etv5* and *Id2*, are located in both the proximal and distal regions of the epithelium at *E*11.5 (Figures 3b and S11–S13). The expected distal localization of the *Sox9* ^+^ cluster is observed starting at *E*12.5 and *E*13.5 (Figures 3b and S11–S13). To validate the spatial position of the proximal and distal epithelial clusters, we profiled the expression of *Sox2*, *Sox9*, and *Shh* (a marker of the entire epithelium) in lung sections using fluorescence in situ hybridization. Our results confirmed that the proximal epithelium contains *Sox9^+^* epithelial cells at *E*11.5, which gradually diminish from this region at later time points (Figures 3c and S14). Quantifying the levels of *Sox2* and *Sox9* within *Shh* ^+^ epithelial pixels revealed that *Sox9* is expressed in a subset of *Sox2^+^* pixels, the fraction of which decreases from *E*11.5 to *E*13.5 (Figures S14 and S15a). Analysis of scRNA‐seq reference data also uncovered a small population (6‐10%) of *Sox2* ^+^ *Sox9* ^+^ cells from *E*11.5 to *E*13.5 (Figure S15b). To determine whether this transcriptional overlap is also reflected at the protein level, we performed wholemount immunofluorescence staining of *E*11.5 lungs, which revealed low but detectable SOX9 protein expression within the proximal epithelium (Figure S15c). Together, these results support the presence of a transient *Sox2* ^+^ *Sox9* ^+^ epithelial population during early lung development. While *Sox2* ^+^ *Sox9* ^+^ epithelial cells have been previously observed in the embryonic human lung, they have not been reported in the mouse, to our knowledge [42]. This discrepancy may reflect differences in sampling strategies, as prior studies largely concentrated on the distal epithelium and later developmental stages [6, 7, 43]. We note that the *Sox2* ^+^ *Sox9* ^+^ epithelium observed here in the mouse lung likely arises through a distinct mechanism from the SOX2^+^SOX9^+^ epithelium reported in the human lung, which has been attributed to distal SOX2 expression rather than the proximal *Sox9* expression found here.

We next subclustered the epithelial cell populations to identify possible transition states between the *Sox2* ^+^ and *Sox9* ^+^ cells (Figure 3d,e and Figure S16a–c). This analysis revealed spatially distinct epithelial subclusters, including Proximal, Proximal Intermediate (PI), Distal Intermediate (DI), and Distal, located along the proximodistal axis of the epithelial tube (Figure 3e and Figure S16a–c). These subclusters show gradients of *Sox2* and *Sox9* markers as well as other markers including *Igfbp5*, *Ccnd2*, *Etv5*, *Id2*, *Bmp4*, and *Sftpc* (Figure 3f and Figure S16d). This spatial and gene expression gradient is consistent with recent findings on airway epithelium during the perinatal and postnatal stages [44]. There is a shift in the composition of these subclusters over time: PI and DI states are observed primarily at *E*11.5 and transition to proximal and distal epithelial cells at *E*12.5 and *E*13.5 (Figure 3g).

To identify genes involved in the initial commitment towards the proximal or distal fates, we analyzed the genes that are highly expressed in both PI and DI (Figure 3h,i and Figure S17a–c). We found that *Smoc2* is upregulated in both PI and DI compared to the other subclusters (Figure 3h,i and Figure S17a–c) and spatial maps showed that *Smoc2* is specifically expressed at high levels in the epithelium immediately adjacent to the proximal and distal‐most ends of the epithelial tubes (Figure 3j). We confirmed the spatial pattern of *Smoc2* and another intermediate‐state marker, *Cldn10*, using in situ hybridization. Again, we used *Shh* to mark the epithelium, and our results showed that both *Smoc2* and *Cldn10* are expressed in the intermediate regions of the epithelial tree (Figure S18). *Smoc2* is more specific than *Cldn10*, as we find a low level of *Cldn10* expression in both the proximal and distal epithelium, which lacks *Smoc2* (Figure S18a,b, zoom‐in; Figure S18c). This observation is consistent with our differential gene expression analysis (Figure 3i). *Adamtsl1*, upregulated in Proximal Epithelium (Figure 3i), is localized to the proximal side (Figure 3k and Figure S17c), while *Bmp4*, *Adamts18*, and *Thbd*, which are highly expressed in Distal Epithelium (Figure 3f and Figure S17c), are at the distal tips (Figure S19). *Bmp4*, *Adamts18*, and *Etv4* have previously been shown to be critical for distal alveolar differentiation, but the exact function of *Smoc2* and *Adamtsl1* in epithelial differentiation requires further investigation [45, 46, 47]. Notably, *Icam1*and *Fgfbp1*, which have been used as markers for intermediate epithelium, were either not identified as a specific marker (*Icam1*) or barely enriched in our analysis (*Fgfbp1*) (Figure 3h and Figure S17a,b,d). We also built a transcriptional pseudotime axis that showed the progression between Proximal and Distal epithelial subclusters (Figure S17e–g). The regression analysis and module‐enrichment analysis along the pseudotime axis revealed similar gene‐expression enrichment in each state (Figure S17h). Altogether, our spatial mapping highlights spatiotemporal dynamics of epithelial transitional states and suggests possible markers of the intermediate states between proximal and distal epithelium.

### 2.4 Spatial Mapping Reveals Matrix Mesenchyme That Localizes Near Neurons and is Enriched in Genes Driving Innervation

**FIGURE 4:**
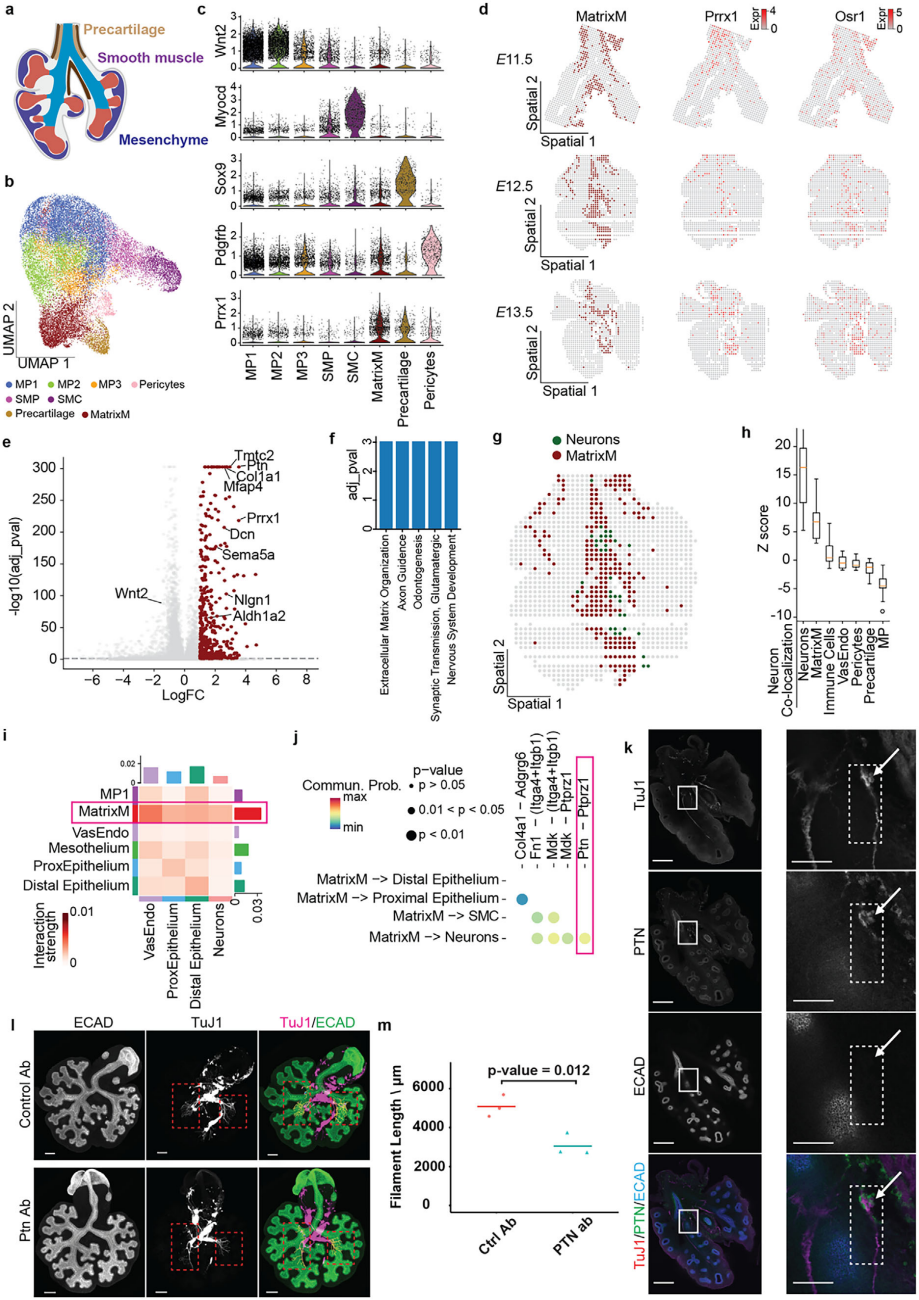
Spatial profiling of Matrix Mesenchyme reveals ECM enrichment and roles in lung innervation. (a) Schematic of mesenchyme within embryonic mouse lung. (b) UMAP of mesenchymal subclusters. (c) Violin plots of top gene markers for each mesenchymal subcluster in scRNA‐seq data. (d) Color‐coded maps of Matrix Mesenchyme (MatrixM), together with spatial maps of the markers *Prrx1* and *Osr1* at each stage. (e) Volcano plot of differential gene expression analysis between Matrix Mesenchyme and the other cell types using scRNA‐seq data. (f) Gene Ontology analysis for the top‐enriched genes in Matrix Mesenchyme. (g) Color‐coded map of Neurons and Matrix Mesenchyme. (h) Z‐score plot for neighborhood‐enrichment analysis of Neurons. MatrixM: Matrix Mesenchyme; VasEndo: Vascular Endothelium; MP: Mesenchymal Progenitors. (i) Heatmap of communication probability between different cell types. ProxEpithelium: Proximal Epithelium. (j) Top ligand‐receptor interactions between Matrix Mesenchyme and other cell types. SMC: Smooth Muscle Cells. (k) Immunofluorescence analysis for TuJ1, PTN, and E‐cadherin for a whole lung section (left) and zoom‐in view (right). Scale bars: 200 µm (left), 50 µm (right). (l) Immunofluorescence analysis for E‐cadherin and TuJ1 in lung explants cultured in the presence of anti‐PTN or control antibodies. The filaments identified in Imaris are highlighted in yellow and enclosed within red dotted boxes. Scale bars: 100 µm. (m) Graph showing quantification of neuronal filaments in lung explants. N = 3.

To study the highly heterogeneous embryonic lung mesenchyme, we isolated the mesenchymal cell types and subclustered the dataset. This analysis revealed eight subclusters, including both mesenchymal progenitors and mature mesenchymal states (Figure 4a–c and Figure S20a). We first focused on the three progenitor states that express high levels of *Wnt2*: Mesenchymal Progenitor 1 (MP1) is in the periphery of the lung lobes and observed at all time points; Mesenchymal Progenitor 2 (MP2) is in the periphery of the lobes as well but first appears at *E*12.5; and Mesenchymal Progenitor 3 (MP3) is close to the vascular endothelium and appears at *E*12.5 and *E*13.5 (Figure S20b). The intermediate and mature cell states in our dataset include Smooth Muscle Progenitors (SMP; *Wnt2* ^low^ *Myocd* ^low^ *Ptch1* ^high^) and Smooth Muscle (SMC; *Myocd* ^high^), both surrounding the epithelium, Precartilage (*Sox9* ^high^) surrounding the trachea and upper airways, and Pericytes (*Pdgfrb* ^high^) adjacent to vascular endothelial cells (Figure 4c and Figure S20a,b). Additionally, we identified a population that we label as Matrix Mesenchyme (MatrixM; *Prrx1* ^high^ *Ptn* ^high^ *Col1a1* ^high^) due to its high expression of ECM proteins and related factors (Figure 4b,c and Figure S20a).

Given the presence of both progenitor and mature smooth‐muscle‐cell states in our dataset, we proceeded to examine the spatial dynamics of mesenchymal progenitors as they differentiate into smooth muscle cells, a process that has been well described using clonal lineage tracing and scRNA‐seq [8, 16, 34, 35, 48, 49, 50]. We reasoned that MP1 is the progenitor source for all mesenchymal cells due to its prevalence at *E*11.5 compared to MP2 and MP3. We then subset the data to include the subclusters of interest and constructed a pseudotime trajectory starting from MP1, through Smooth Muscle Progenitors, and into Smooth Muscle (Figure S20c,d). To test whether our dataset could provide spatial context for the differentiation trajectories of the mesenchymal subclusters, we mapped the pseudotime analysis spatially. We observed a spatial association between smooth muscle maturation and lung topology: the most immature MP1 localizes to the periphery of the lung, while Smooth Muscle Progenitors and Smooth Muscle cells progressively align closer to the bronchi along the distal‐to‐proximal axis (Figure S20c,d). This spatial pattern aligns with previously published observations that mesenchymal progenitors adjacent to epithelial tips are recruited to the sites of branching to differentiate into smooth muscle [8, 51].

We next focused on the Matrix Mesenchyme population, which expresses high levels of several ECM‐related genes, including *Col1a1*, *Tmtc2*, *Col1a2*, and *Col3a1*, as well as markers *Ptn*, *Prrx1*, and *Osr1* (Figure 4d,e and Figures S20a,S21a). Notably, previous studies identified this population of cells but grouped it together with mesenchymal progenitors [34, 36]. While it shares some genes with mesenchymal progenitors, such as those related to collagen, Matrix Mesenchyme expresses minimal levels of the mesenchymal progenitor marker *Wnt2* (Figure 4c, Figure S20a) [36, 52, 53]. Our spatial dataset revealed that Matrix Mesenchyme is consistently located around the trachea and along the medial boundaries of the lobes, distinct from *Wnt2* ^high^ Mesenchymal Progenitors that localize to the lung periphery (Figure 4d and Figure S20b). To verify the spatial location of these cells, we conducted fluorescence in situ hybridization analysis for *Prrx1*, *Osr1*, and *Sox9*, together with DAPI staining for cell segmentation. We used *Sox9* to distinguish between Precartilage (*Sox9* ^high^) and Matrix Mesenchyme (*Sox9* ^low^), as Precartilage also expresses both *Prrx1* and a low level of *Osr1*, and localizes to the trachea (Figure 4c and Figure S21b). We observed a spatial pattern of *Prrx1* ^high^ *Osr1* ^high^ *Sox9* ^low^ cells, consistent with our spatial dataset (Figure 4d and Figure S22). Similarly, we also performed fluorescence in situ hybridization analysis for *Prrx1*, *Col1a1*, and *Ptn* and our results revealed co‐expression of these markers in cells in both trachea and carina of the lung, corresponding to the Matrix Mesenchyme (Figure S23). We therefore conclude that Matrix Mesenchyme is a distinct population of cells in the embryonic mouse lung and likely does not function as a mesenchymal progenitor.

To investigate the potential function of Matrix Mesenchyme, we performed Gene Ontology (GO) analysis on the most upregulated genes in this population compared to all other mesenchymal cells (Figure 4e,f). As expected from its high expression of ECM‐related genes, Matrix Mesenchyme shows strong enrichment in gene sets associated with Extracellular Matrix Organization (Figure 4f, *p*  = 9.3 × 10^−4^). Matrix Mesenchyme is also enriched in innervation‐related terms, including Axon Guidance (*p* = 9.3 × 10^−4^), Synaptic Transmission, Glutamatergic (*p* = 9.3 × 10^−4^), Nervous System Development (*p* = 9.3 × 10^−4^), and Neuron Projection Guidance (*p* = 9.3 × 10^−4^), suggesting a potential function in the development and patterning of the lung nervous system (Figure 4f).

Consistent with this hypothesis, neighborhood‐enrichment analysis revealed that Matrix Mesenchyme and Neurons are frequently located in close spatial proximity to each other (Figure 4g,h and Figure S24a–c). To tease out the potential effect of adjacent esophageal tissue on the Matrix Mesenchyme and its proximity to neuron populations, we removed pixels in the medial part of lung lobes and adjacent pixels close to the trachea and performed neighborhood‐enrichment analysis again on the remaining spatial data (Figure S25a,b). We found that these removed pixels indeed had a high proportion of Matrix Mesenchyme but our neighborhood‐enrichment analysis on the cleaned dataset still showed significant enrichment between the remaining Matrix Mesenchyme and Neurons, suggesting a potential interaction between these two cell populations (Figure S25c,d).

Supporting this idea, cell‐cell communication analysis using cellchatV2 identified significant ligand‐receptor interactions between Matrix Mesenchyme and Neurons (Figure 4i) [54, 55]. Among these predicted signaling pathways, one notable example is the PTN‐PTPRZ1 ligand‐receptor axis (Figure 4j). *Ptn* encodes for pleiotrophin (PTN), a multifunctional growth factor that has been reported to promote the migration and neurite outgrowth of embryonic neurons [56, 57]. In our dataset, *Ptn* is highly expressed in Matrix Mesenchyme (Figure S24d,e), while two known PTN receptors, *Sdc3* and *Ptprz1*, are both specifically expressed in Neurons at these stages of lung development (Figure S24f). Immunofluorescence analysis further revealed that PTN‐expressing cells are positioned adjacent to neuronal cells marked by class III beta‐tubulin (TuJ1) but are largely absent from epithelial cells marked by E‐cadherin (Figure 4k and Figure S26). To confirm these PTN‐expressing cells and associated neurons do not represent contamination from the esophageal tissue, we stained for WT1 together with PTN and TuJ1. While no distinct mesothelial boundary was observed at the medial edge of the lung lobes, the PTN+ cells in this region were clearly distinguishable from esophageal tissue (highlighted cells, Figure S27a,b). Similarly, no clear mesothelial boundary was observed in the trachea, where PTN+ cells lie proximal to neuronal cells (Figure S27b). The lack of mesothelial boundary in the carina and trachea is maintained at *E*13.5 (Figure S27c). Our observation is consistent with previous reports that WT1+ cells are absent from both surfaces of the carina and the dorsal surface of the trachea at comparable embryonic stages [58, 59, 60]. We reason that the local absence of mesothelium in these regions may facilitate neuronal migration from the esophagus to the lung, though this possibility requires further validation.

To determine the role of PTN in neuronal patterning, we cultured embryonic lung explants in the presence of a PTN function‐blocking monoclonal antibody, which led to a significant reduction in the presence of neuronal filaments innervating bronchi (Figure 4l,m and Figure S28, *p* = 0.012, see Methods). Together, these data suggest that Matrix Mesenchyme forms a specialized mesenchymal niche that supports early lung innervation through multiple signaling pathways, including downstream of PTN. Our spatial mapping using the Enhanced DBiT‐seq workflow thus identifies Matrix Mesenchyme as a previously unrecognized mesenchymal subcluster with distinct gene expression and spatial locations, which may promote lung innervation during early stages of lung development.

## 3 Discussion

Here, we optimized DBiT‐seq and demonstrated improved transcript recovery compared to existing whole‐transcriptomic spatial transcriptomics methods that rely on mRNA diffusion. Microfluidics‐based approaches such as DBiT‐seq enhance detection sensitivity by delivering spatial DNA barcodes directly into the tissue, bypassing the need for mRNA to diffuse to capture probes [23]. By optimizing tissue fixation, incorporating random‐primed second‐strand synthesis, and relocating the RT step to a bulk reaction volume, we further improved detection sensitivity. Collectively, these improvements doubled UMI recovery on average and increased gene detection while maintaining 10‐µm spatial resolution.

We tested whether our optimized spatial transcriptomics protocol could uncover the spatial dynamics of key cell states by using the embryonic mouse lung as a model. Our spatial analysis confirms epithelial differentiation along the proximodistal axis of the organ but surprisingly reveals that *Sox9* ^+^ *Etv5* ^+^ *Id2* ^+^ epithelial progenitors are localized in proximal regions of the epithelial tree at *E*11.5. This unexpected spatial pattern contrasts with the prevailing conceptual model of lung development, which assumes an initial proximodistal patterning of the epithelial tree, with distal epithelial cells serving as the sole progenitors for different mature epithelial cell types [1, 2, 5]. Nevertheless, our findings reconcile previous lineage‐tracing studies showing that *Id2* ^+^ progenitors, located both proximally and distally at *E*12.5 in our spatial data (Figure S12d–f), give rise to both bronchiolar and alveolar cell types when labeled before *E*12.5 but only alveolar cell types when labeled at *E*16.5 [5]. Additional studies using precisely time‐controlled lineage tracing are needed to validate the differentiation trajectories of the epithelium at different time points. In addition, we observed co‐expression of *Sox2* and *Sox9* in subsets of the epithelium at *E*11.5, a phenomenon that has previously been reported in the embryonic human lung but not the mouse.

Our optimized pipeline was especially useful for disentangling the heterogeneous mesenchymal compartment. We uncovered a previously undescribed mesenchymal cluster, Matrix Mesenchyme, which localizes near the trachea and medial boundaries of the lobes and is characterized by high expression of ECM‐related proteins. While this cluster is present in scRNA‐seq reference data, it was previously classified as part of the mesenchymal progenitor population [34, 36]. Two lines of evidence support our conclusion that Matrix Mesenchyme represents a distinct cell type. First, Matrix Mesenchyme expresses low levels of *Wnt2* and markers of proliferation, which are expressed at high levels in mesenchymal progenitors. Second, Matrix Mesenchyme maps to locations that are distinct from mesenchymal progenitors, which localize to the periphery of the lung. We also predicted multiple interactions between Matrix Mesenchyme and Neurons. Among these, the PTN signaling axis emerged as a notable candidate. Lung explant culture experiments blocking PTN signaling reduced neuronal filament length, consistent with a potential role for Matrix Mesenchyme in innervation of the early embryonic lung. We want to note that while Matrix Mesenchyme shows the highest expression level of *Ptn*, this marker is also expressed by other cell types, including mesenchymal progenitors, precartilage, pericytes, and neurons themselves (Figure S24d). Given that the signals that promote migration of intrinsic neurons into the lung remain unclear, we postulate that spatial transcriptomics approaches can help fill the gaps by pinpointing the critical ligand‐receptor interactions between neuronal progenitors and other cell types [25, 61, 62, 63].

While spatial mapping of scRNA‐seq clusters facilitates the investigation of the epithelial intermediate subcluster and heterogeneous mesenchymal populations, identifying differentially expressed genes in pixels that are spatially distinct will also enable the discovery of spatially variable genes. To that end, we annotated obvious regions of domain branching and bifurcation within *E*11.5 and *E*12.5 lung sections (Figure S29a–c). We then performed differential gene expression analysis for Epithelium, Mesenchymal Progenitors, and Mesothelium, respectively. We found no significant difference between the different branching modes for Epithelium and Mesothelium, while we observed Mesenchymal Progenitors in bifurcation sites are highly enriched in *Snai2* (Figure S29d). *Snai2* is a known regulator of ECM‐related genes but the association between this transcription factor and branching morphogenesis of the lung requires further investigation.

It is important to note that the gene‐expression pattern of Matrix Mesenchyme closely resembles that of Alveolar Fibroblast 2 (AF2), a cell type previously identified in the Mouse Lung Cell Atlas, except that *Mfap5*, a top marker of AF2, is not enriched in Matrix Mesenchyme (Figure S30) [64]. The similarity between Matrix Mesenchyme and AF2 is unexpected, as AF2 is primarily observed postnatally, while our data are limited to early embryonic stages [3, 36, 65, 66]. Moreover, the distinct locations of each cell type suggest that Matrix Mesenchyme has a different function than AF2 at this early stage. Future studies are needed to determine how Matrix Mesenchyme matures at later stages and to clarify its relationship with AF2.

To link our mesenchymal subclusters with previous studies, we note that MP1 has a similar gene‐expression profile as subepithelial mesenchyme, whereas MP2 and Matrix Mesenchyme resemble submesothelial mesenchyme [35]. However, our subclustering in this study resulted in finer resolution with additional spatial context.

Our study has limitations. First, we acknowledge that cellular purity at each spatial spot/pixel remains a limitation shared by current sequencing‐based spatial transcriptomics technologies, including the platform used in this study. Because binning or pixel‐based capture aggregates transcripts from multiple cells, spots located at cell boundaries or in densely packed tissue regions may contain mixed transcriptomic signals from more than one cell type. Technologies that achieve single‐cell resolution without pixel binning, such as Slide‐tags, are in principle less susceptible to this issue. Rigorously benchmarking cellular purity across platforms would require profiling serial sections with each technology alongside matched scRNA‐seq data for deconvolution, which is beyond the scope of the current study. Nonetheless, higher‐resolution methods (e.g., Visium HD, Stereo‐seq) are expected to achieve better cellular purity within their binned spots compared with lower‐resolution technologies, and we have taken this into consideration when interpreting our spatial data by performing all differential gene‐expression analyses using scRNA‐seq data to avoid any confounding issues. Another limitation is that the top markers of Matrix Mesenchyme are not specific, including *Ptn* and *Prrx1*, making it challenging to deconvolve how other cell types contribute to lung innervation via the PTN‐PTPRZ1 signaling pathway. The roles of other gene markers driving innervation terms remain to be investigated. As we were preparing this manuscript, a similar spatial mapping of embryonic lungs using the original DBiT‐seq protocol was published [67]. However, that work focuses on more mature embryonic stages and lacks the insight we have on the early embryonic stages, likely due to limited resolution (20 µm) and/or detection sensitivity. Our optimized spatial transcriptomic mapping shows a superior efficiency of transcript recovery at high resolution, which allowed us to reveal the early spatiotemporal pattern of epithelial progenitors and identify mesenchymal subpopulations with distinct distributions and developmental roles, providing a valuable resource for studying lung morphogenesis and innervation.

## 4 Experimental Section/Methods

### 4.1 Mice

Embryos were isolated from euthanized timed‐pregnant mice (*Mus musculus*, C57BL/6J) at *E*11.5–13.5 and stored in chilled phosphate‐buffered saline (PBS) prior to dissection. Lungs were dissected following standard protocols in chilled PBS supplemented with 10‐U mL^−1^ penicillin and 10‐µg mL^−1^ streptomycin (Sigma‐Aldrich). All procedures involving animals were approved by Princeton University's Institutional Animal Care and Use Committee (IACUC Protocol #1855). Mice were housed in an AAALAC‐accredited facility in accordance with the NIH Guide for the Care and Use of Laboratory Animals. This study was compliant with all relevant ethical regulations regarding animal research.

### 4.2 Embedding and Sectioning

To embed samples, the dissected lungs were incubated for 1 h on a shaker at 4°C in 0.3 mL each of 20% sucrose, 30% sucrose, and 1:1 OCT:30% sucrose solution with 0.6‐µL DEPC, sequentially, before embedding in disposable base molds using dry ice. The embedded lungs were warmed up at −20°C in a cryostat and sectioned into ∼7‐µm thickness before being placed in the center of poly‐L‐lysine‐coated glass slides. The cryosections were stored at ‐80°C before testing. In total, we collected 5 sections from five *E*11.5 embryonic lungs, 4 sections from three *E*12.5 embryonic lungs (2 out of 4 sections are from a single embryonic lung), and 4 sections from four *E*13.5 embryonic lungs.

### 4.3 Immunostaining

Lungs were dissected and fixed in 4% paraformaldehyde (PFA) in PBS on a shaker at 4°C for 15 min. Lungs were then washed four times for 15 min with 0.5% Triton X‐100 in PBS and blocked overnight at 4°C using 5% donkey serum in PBST on a shaker. Samples were then incubated with primary antibodies against PTN (mouse, 1:100, Santa Cruz Biotechnology sc‐74443), E‐cadherin (rat, 1:400, Invitrogen 131900), TuJ1 (rabbit, 1:400, R&D Systems MAB1195‐SP, or chicken, 1:200, Novus Biologicals NB100‐1612), Sox2 (goat, 1:200, R&D Systems AF2018), Sox9 (rabbit, 1:500, EMD Millipore AB5535), WT1 (rabbit, 1:100, Abcam ab89901) for three days (*E*12.5 and explants) or two days (*E*11.5) at 4°C on a shaker, followed by Alexa Fluor‐conjugated secondary antibodies (1:200 for Thermo Fisher Scientific A21121; 1:400 for Thermo Fisher Scientific A21245, A48263, A78946, A32788, Biotium 20015; 1:500 for Thermo Fisher Scientific A78947, A32816, A32790) and Hoechst (1:1000) for another 2 days at 4°C.

### 4.4 Lung Explant Culture and Neuronal Filament Quantification

Lungs (*Mus musculus*, CD1) were dissected at early *E*11.5 with special care to preserve the structure of the trachea and the regions between medial boundaries of lung lobes, in order to maintain neuronal tissues as intact as possible. Lung explants were then cultured as previously described [68]. Briefly, dissected lung explants were positioned on top of a porous membrane (nucleopore polycarbonate track‐etch membrane, 8‐µm pore size, Whatman), floating on the surface of DMEM/F12 medium supplemented with 50 units mL^−1^ of penicillin and streptomycin, 5% fetal bovine serum (FBS, heat inactivated; Atlanta Biologicals), containing either desalted anti‐PTN antibody (6 µg mL^−1^; Santa Cruz Biotechnology sc‐74443) or mouse IgG1 antibody (Mouse IgG1 Isotype Control, Invitrogen, 026100) as a control. Lungs were cultured for 36 h, then fixed in 4% PFA for 15 min at 4°C, followed by immunostaining. We note that the staging of the embryonic lungs and the culture duration are critical for this inhibition experiment.

We then used Imaris (v10.2.0) to quantify the neuronal filaments in the cultured lungs. Filament structures were reconstructed using the Tree AutoPath Algorithm to define neuronal segments and identify filaments. The estimated largest diameter for starting points was set to 16 µm, while the thinnest diameter was set to 0.958 µm. To minimize the effect of variability in the initial neuronal cell number introduced during dissection, analysis was restricted to neuronal filaments innervating the right and left lung lobes (see highlights in the boxed regions in Figure 4l and Figure S28), and the total filament length was measured. We then performed a two‐tailed t‐test to compare the total filament length across different conditions.

### 4.5 Fluorescence In Situ Hybridization

Adjacent lung sections were used for fluorescence in situ hybridization to validate the spatial transcriptomic mapping. Specifically, we used the RNAscope analysis Multiplex Fluorescent V2 Assay (ACD) protocol. Probes used were *Mus musculus Shh* (314361‐C1), *Sox9* (401051‐C3), *Sox2* (401041‐C2), *Prrx1* (485231‐C1), *Osr1* (496281‐C2), *Smoc2* (318541‐C2), *Cldn10* (541041‐C3), *Col1a1*(319371‐C2), and *Ptn* (486381‐C3). Sections were imaged on a spinning disk confocal (BioVision X‐Light V2) fitted to an inverted microscope.

### 4.6 Microfabrication

Our spatial tests used two microfluidic devices – Chip A for loading barcodes A1‐A50 and Chip B for loading barcodes B1‐B50 in an orthogonal direction, following a design from previous studies [23]. Master molds were microfabricated via standard photolithography on 4‐inch silicon wafers. Specifically, a layer of SU8‐2025 photoresist (∼25 µm) was spin‐coated and patterned using the Heidelberg DWL66+ direct write lithography system prior to developing. The mold was hard baked at 150°C and silanized for 15 min before device fabrication. Chips A and B were fabricated by adding ∼30‐g 10:1 (base to curing agent ratio) Sylgard 184 (Dow Corning 4019862) onto the mold and curing for 2 h at 65°C. The inlets and outlets of each device were then punched and cleaned using IPA for 10 min with sonication before use.

### 4.7 Spatial Sequencing Using Enhanced_DBiT_v1

#### 4.7.1 Preprocessing

Sections were taken from the freezer and imaged at the desired optical resolution (4×, 10×, or 20× air objective). The sections were fixed using a mixture of 800‐µL ice‐cold methanol, 15‐µL 50‐mg mL^−1^ DSP in dimethyl sulfoxide, and 1.6‐µL diethyl pyrocarbonate for 15 min. The sections were then cleaned by pipetting 1 mL of PBS‐RI (998.6 µL PBS with 1.4 µL RNase inhibitor) across the tissue three times and then dried with gentle air flow.

The tissue sections were then blocked for 30 min in 100‐µL BSA (10 mg mL^−1^) in PBS at 4°C in a humidified chamber, followed by washing three times with 100‐µL 0.5x PBS‐RI for 5 min, followed by another two quick washes using 0.5x PBS‐RI.

To start spatial barcoding, the first microfluidic device was centered on the tissue. We then took a scan to record the position of the channels on the tissue. We added and loaded 1‐mL PBST into the reservoir to permeabilize the tissue section for 20 min. We then removed the remaining PBST from the reservoir and the inlets/outlets and washed the channels by adding ∼1‐mL 0.5x PBS‐RI to the reservoir and flowing for about 10 min. We applied vacuum for 5 min to remove any remaining washing buffer from the channels.

#### 4.7.2 Reverse Transcription

We first prepared the following RT mixture: 29.2‐µL RNase‐free water, 55‐µL 5×Maxima RT buffer, 110‐µL 0.5×PBS‐RI, 1.76‐µL RNase inhibitor (Enzymatic), 3.52‐µL Superase In RNase Inhibitor (Ambion), 13.75‐µL 10‐mM dNTPs, 27.5‐µL Maxima H Minus Reverse Transcriptase, and 6.88‐µL 100‐µM template switch oligos (Table S1). We then combined 4.5‐µL RT mixture and 0.5 µL of 25‐µM Barcode A1‐A50 (Table S1), before introducing the barcode mixture into the inlets of the microfluidic device sequentially. We then applied the vacuum to fill all channels with solution and incubated in a humidified chamber at room temperature for 30 min followed by 42°C for 90 min for in‐tissue reverse transcription.

Following reverse transcription, we removed the barcode A mixture from the inlets/outlets and cleaned the channels by flushing with 0.5‐mL NEB buffer 3.1 with 1% Enzymatics RNase Inhibitor (10 µL for each inlet) for 10 min. We then peeled off Chip A and briefly dipped the slide in a 50‐mL Falcon tube with RNase‐free water and gently dried under air.

#### 4.7.3 Ligation

We then placed and centered Chip B on the tissue, imaging the channels to ensure proper placement. After firmly clamping Chip B on the tissue section, we prepared the ligation mixture as follows: 55.39‐µL RNase‐free water, 27.5‐µL 10× T4 ligase buffer, 11.2‐µL T4 DNA ligase (400 U µL^−1^), 2.24‐µL RNase inhibitor (Enzymatics), 0.71‐µL Superase In RNase Inhibitor (Ambion), 5.5‐µL 5% Triton‐X‐100, and 117.9‐µL 1× NEB buffer 3.1 with 1% RNase inhibitor (Enzymatics). We combined 4‐µL ligation buffer with 1‐µL 25‐µM barcode B1‐B50 (Table S1), before introducing the barcode B buffer into the channels sequentially. We then incubated in a humidified chamber at 37°C for 30 min for in‐tissue ligation of barcode Bs. Following ligation, we cleaned the channels by flushing with wash buffer (500‐µL PBS, 5‐µL 10% Triton X‐100, 1.25‐µL Superase In RNase Inhibitor) for 10 min. We then peeled off Chip B and dipped the slide into a 50‐mL falcon tube with RNase‐free water before gently drying under air. We took a final image of the tissue section to enable mapping and alignment of the sequencing results.

#### 4.7.4 Lysis and Sub‐Library Generation

To collect tissue‐derived cDNA, we added 10 or 15 µL of lysis buffer (0.5x PBS, 10‐mM Tris pH 8.0, 200‐mM NaCl, 50‐mM EDTA pH 8.0, 2.2% SDS, 2‐mg mL^−1^ Proteinase K) for 10‐µm and 15‐µm tests, respectively, to the spatially barcoded tissue sections. We then incubated the tissue section at 55°C for 2 h in a tightly sealed and humidified chamber. We collected the lysate into a 1.5‐mL DNA low‐binding centrifuge tube and then added the same amount of extra lysis solution to wash out the reservoir and retrieve as much lysate as possible. The cell lysate was immediately stored at −80°C.

#### 4.7.5 Cell lysate Purification

To purify cDNA from cell lysates, we first prepared 40‐µL Dynabeads MyOne Streptavidin C1 beads (Thermo Fisher) per sample, following the supplier's instructions. We then added 7 volumes of DNA Binding Buffer to each volume of cDNA sample before mixing briefly by vortexing. We purified the mixture using the provided Zymo‐Spin Column in a Collection Tube. After two washes, we eluted the purified cDNA using 100‐µL RNase‐free water. We then further captured biotin‐labelled cDNAs by mixing 100 µL of resuspended Dynabeads MyOne Streptavidin C1 magnetic beads to each lysate at room temperature for 60 min. We then washed the C1 beads twice with 400 µL of B&W buffer (2.5‐mM Tris‐HCl, pH 8.0, 0.5‐M NaCl, 0.25‐mM EDTA, pH 8.0) with 5 min rotation after resuspending beads, followed by another wash once with 400‐µL 10‐mM Tris‐HCl with Tween‐20 with 5 min rotation after resuspension.

#### 4.7.6 Random‐Priming‐Assisted Second‐Strand Synthesis

The beads with cDNA captured were first resuspended in 200 µL of ExoI mix [170‐µL water, 20‐µL ExoI buffer, and 10‐µL ExoI (NEB, M0568)] and incubated at 37°C for 50 min. Following ExoI treatment, the beads were washed three times using 400 µL of B&W buffer with 0.05% Tween (2.5‐mM Tris‐HCl, pH 8.0, 0.5‐M NaCl, 0.25‐mM EDTA, pH 8.0, 0.1% Tween‐20). The bead pellet was then resuspended in 200 µL of 0.1‐M NaOH and incubated for 5 min at room temperature. Subsequently, 400 µL of B&W buffer was added and the supernatant was then discarded, and the beads were washed three times in 400 µL of B&W buffer with 0.05% Tween.

The random‐priming‐assisted second‐strand synthesis was then carried out on the beads by incubating them in 200 µL of second‐strand synthesis mix (2‐µL 1‐mM dN‐SMRT oligo (Table S3), 5‐µL Klenow enzyme, 20‐µL 10‐mM dNTPs, 40‐µL 5x Maxima RT buffer, 133‐µL RNase‐free water) at 37°C for 1 h. After the reaction, the supernatant was removed, and the beads were washed three times using 400 µL of B&W buffer with 0.05% Tween‐20.

#### 4.7.7 PCR

The beads were then washed once with 400 µL of 10‐mM Tris and 0.1% Tween‐20 solution (with resuspension) and once more with 400 µL of RNase‐free water (without resuspension). The beads were then resuspended in the 220‐µL PCR mix solution (110‐µL 2x Kapa HiFi HotStart Master mix, 8.8‐µL 10‐µM Primers (Table S3), and 92.4‐µL RNase‐free water). The PCR cycles were as follows: 95°C for 3 min, followed by 5 cycles at 98°C for 20 s, 65°C for 45 s, and 72°C for 3 min. The beads were then removed, and the remaining supernatant was used to run the second PCR: 95°C for 3 min, followed by 10–15 cycles at 98°C for 20s, 65°C for 20s, and 72°C for 3 min, followed by 72°C extension for 5 min. We then purified cDNA from the PCR product using 0.8x KAPA pure beads following the supplier's instructions.

#### 4.7.8 Sequencing

The sequencing library was then prepared with custom P5 primer (Table S3) using a Nextera XT DNA Library Preparation Kit by following the supplier's instructions. The samples were sequenced using Nova‐seq SP 300nt Flowcell with 80 bp for read1, and 250 bp for read 3.

### 4.8 Spatial Sequencing Using Enhanced_DBiT_v2

As most steps are similar to those described for Enhaced_DBiT_v1, only the major modifications are highlighted here.

#### 4.8.1 Reverse Transcription

After the tissue sections were blocked and washed, reverse transcription was performed using a 5‐mm‐wide PDMS reservoir. Specifically, the following reaction mixture was added onto each tissue section: 11.3‐µL RNase‐free water, 12.5‐µL 5× Maxima RT buffer, 25‐µL 0.5× PBS‐RI, 0.4‐µL RNase inhibitor (Enzymatic), 0.8‐µL Superase In RNase Inhibitor (Ambion), 3.1‐µL 10‐mM dNTPs, 6.2 ‐µL Maxima H Minus Reverse Transcriptase, 1.6‐µL 100‐µM template switch oligos, and 1.6‐µL 100‐µM RT primer (Table S2). Tissue sections were incubated in a humidified chamber at room temperature for 30 min, followed by 42°C for 90 min for in‐tissue reverse transcription.

Following reverse transcription, the reaction mixture was removed from the reservoir, and the tissue section was washed with 150‐µL NEB buffer 3.1 with 1% Enzymatics RNase Inhibitor for 5 min. The PDMS reservoir was then peeled off, and the slide was briefly dipped in a 50‐mL falcon tube with RNase‐free water and gently dried under air.

#### 4.8.2 Ligation

Chip A was then aligned and centered on the tissue section, and channel placement was confirmed by imaging before Chip A was firmly clamped on the tissue section. At the same time, the ligation mixture was prepared as follows: 65.45‐µL RNase‐free water, 32.5‐µL 10× T4 ligase buffer, 13.24‐µL T4 DNA ligase (400 U/µL), 2.65‐µL RNase inhibitor (Enzymatics), 0.84‐µL Superase In RNase Inhibitor (Ambion), 6.5‐µL 5% Triton‐X‐100, and 139.38‐µL 1× NEB buffer 3.1 with 1% RNase inhibitor (Enzymatics). For each channel, 4‐µL ligation buffer was combined with 1‐µL 25‐µM barcode A1‐A50_v2 (Table S2), and the barcode A solutions were introduced into the channels sequentially. Slides were incubated in a humidified chamber at 37°C for 30 min for in‐tissue ligation of barcode As. Following ligation, channels were flushed with NEB buffer 3.1 with 1% Enzymatics RNase Inhibitor for 5 min.

Ligation of barcode B1‐B50_v2 oligonucleotides (Table S2) was then performed using Chip B following the same procedure. After ligation, channels were washed for 5 min with wash buffer containing 500‐µL PBS, 5‐µL 10% Triton X‐100, and 1.25‐µL Superase In RNase Inhibitor).

All remaining steps were identical to those described for Enhanced_DBiT_v1.

### 4.9 Computational Methods

#### 4.9.1 DBiT‐seq Processing

Read 1 was reformatted to extract UMI, Barcode A, and Barcode B sequences. The reformatted Read 1 and Read 3 were mapped against the mouse genome (GRCh39), demultiplexed, and annotated (Gencode release M31) using the ST pipeline v1.8.1. The resulting gene‐expression matrix was then filtered by removing pixels that did not cover tissues.

#### 4.9.2 Saturation Analysis

To compare the original DBiT‐seq and Enhanced_DBiT, raw sequencing reads were subsampled from 1M to 25M, and the same data processing workflow described above was applied using ST pipeline v1.8.1 (https://github.com/jfnavarro/st_pipeline) to obtain UMI and gene counts at each sequencing depth for all tissue pixels [23, 69]. To compare Enhanced_DBiT, Slide‐seqv2, Slide‐tags, and Visium, Spacemake was used to process the raw data from each technology and generate aligned sequencing reads [70]. We then followed a previously established computational protocol (https://github.com/rajewsky‐lab/openst/tree/benchmark) to subsample aligned sequencing reads from filtered, randomly selected spatial pixels and generate UMI and gene accounts at each subsampled read depth, with a correction accounting for reads removed during preprocessing step, including spatial barcode matching and non‐coding RNA removal [21].

#### 4.9.3 Transcriptomic Integration

Preprocessed DBiT‐seq replicates were integrated together with the previously published scRNA‐seq reference datasets [34, 35, 36, 37] to provide higher resolution of transcriptomic signals and facilitate cell‐state annotation of the DBiT‐seq datasets. Prior to integration, to eliminate sequencing‐depth‐based batch effects, we subsampled the reference datasets to an average of 20k reads per cell and normalized all datasets using SCTransformV2 [71]. We then integrated the datasets (IntegrateLayers using RPCA and indicating reference datasets), followed by clustering and UMAP calculation (RunUMAP with dims = 1:13). Once completed, the integrated object was visualized via UMAP, and cell‐state annotation was carried out using expression of canonical marker genes of expected lung‐cell states. To verify cell‐state identities, integrated annotations were compared against previous annotations per reference dataset.

#### 4.9.4 Cell Type Deconvolution Using Conditional Autoregressive Model‐Based Deconvolution (CARD)

Cell type deconvolution was performed using CARD (https://github.com/YMa‐lab/CARD) [39]. Briefly, we integrated and built scRNA‐seq reference following a protocol similar to that described above [34, 35, 36, 37]. We then performed CARD deconvolution using the annotated scRNA‐seq reference together with the per‐sample spatial count matrices to obtain predicted cell type proportions for each spatial pixel. Spatial pixels were subsequently filtered using a threshold of 0.5 for maximum predicted cell type proportion. Gene expression correlation analysis between different cell types in scRNA reference was performed using scanpy.

#### 4.9.5 *Sox2* ^+^ *Sox9* ^+^ Epithelial Cell Quantification in scRNA‐seq

Cells were counted as *Sox2* ^+^ *Sox9* ^+^ if the normalized and log‐scaled raw counts of both *Sox2* and *Sox9* were larger than 0.1 for only cells from the scRNA‐seq reference datasets.

#### 4.9.6 Differential Expression and GO Analysis

Using the integrated dataset described above, differential expression analysis was carried out between pairwise cell‐state comparisons using the normalized and log‐scaled raw counts for only cells from the scRNA‐seq reference datasets to avoid technology‐driven batch effects. Differential expression was calculated using the Wilcoxon rank‐sum test offered in the scanpy rank_gene_groups function and the log‐fold change was calculated as the log_2_(Group1 + pseudocount) – log_2_(Group2 + pseudocount) with a pseudocount of 0.01. To calculate enriched gene sets, enriched genes (p<0.05, log fold change > 1.3) were analyzed using Enrichr.

#### 4.9.7 Pseudotime Analysis

Pseudotime analysis was then performed using the Monocle 3 package^74^. Since the integrated dataset contains multiple distinct trajectories, we selected only cell states related to each trajectory prior to generation of the pseudotime axis and subsequent gene‐module analysis. The dataset subsets were selected using primary marker genes and temporal dynamics: the epithelial subclusters for the Epithelial trajectory; Mesenchymal Progenitor 1, Smooth Muscle Progenitor, and Smooth Muscle for the Smooth Muscle trajectory. The previously calculated UMAP, cell‐state assignments, and spatial localization maps were used for visualizing pseudotime trajectories. To characterize gene sets involved in each differentiation trajectory, the Monocle 3 package was used to identify gene modules that co‐vary along the pseudotime axis. First, genes that vary significantly across the full pseudotime axis were identified (graph_test, neighbor_graph = 4) using the Morans I spatial autocorrelation analysis. Then, genes were grouped into modules using Louvain community clustering based on the cells that express them (find_gene_modules). The resulting gene modules were investigated by scoring the average gene expression within different cell‐state categories and individual cells using the scanpy_score_genes function. Additional genes that vary along the pseudotime axis were identified using linear regression analysis using the scipy_linregress function.

#### 4.9.8 Neighborhood‐Enrichment and Localization Analysis

Neighborhood‐enrichment analysis was carried out using the Squidpy [72] package (gr.nhood_enrichment) to measure the neighborhood enrichment of each cell state per each individual dataset.

## Author Contributions

Pengfei Zhang: conceptualization, investigation, funding acquisition, writing – original draft, methodology, validation, writing – review and editing, visualization, data curation, formal analysis, software. Ben Law: investigation, funding acquisition, writing – original draft, methodology, writing – review and editing, visualization, data curation, formal analysis, software. Niles Huang: Validation, Methodology. Katharine Goodwin: Validation, Methodology. Celeste Nelson: supervision, methodology, conceptualization, funding acquisition, project administration, writing – review and editing. Michelle Chan: Supervision, methodology, conceptualization, funding acquisition, project administration, writing – review and editing.

## Funding

This work was supported in part by the National Institutes of Health (NIH) [HD099030, HL164861, HD111539, HD144311 (to C.M.N.), HD111537 (to M.M.C.), and T32GM007388 (to B. L.)] and the National Science Foundation (NSF) (2134935 to C.M.N.).

## Conflicts of Interest

The authors declare no conflicts of interest.

## Supporting information




**Supporting File 1**: advs77678‐sup‐0001‐SuppMat.docx.

**Supporting File 2**: advs77678‐sup‐0001‐SuppMat.xlsx.

## Data Availability

The spatial transcriptomic data are available from the Gene Expression Omnibus (GEO) database under accession numbers GSE331261, GSE331262, GSE331265. https://dataview.ncbi.nlm.nih.gov/object/PRJNA1466050?reviewer=7e1gf63qojunq7vj73bp7obqn3. Accession numbers or link for raw sequencing data from other technologies are as follows: DBiT_seq (GSM4096261, GSM4189615, GSM4364242, GSM4364243, GSM4364244, GSM4364245), Slide‐seqv2 (GSM5915059), Slide‐tags (GSM7814156), Visium (https://s3‐us‐west‐2.amazonaws.com/10x.files/samples/spatial‐exp/2.0.0/V1_Mouse_Brain_Sagittal_Anterior_Section_2/V1_Mouse_Brain_Sagittal_Anterior_Section_2_fastqs.tar). The code supporting the findings of this study is available on GitHub at https://github.com/BenjaminKLaw/Zhang_and_Law_et_al.

## References

[1] E. E. Morrisey and B. L. M. Hogan , “Preparing for the First Breath: Genetic and Cellular Mechanisms in Lung Development,” Developmental Cell 18, no. 1 (2010): 8–23, 10.1016/j.devcel.2009.12.010. PMC3736813 20152174

[2] J. A. Zepp and E. E. Morrisey , “Cellular Crosstalk in the Development and Regeneration of the Respiratory System,” Nature Reviews Molecular Cell Biology 20, no. 9 (2019): 551–566, 10.1038/s41580-019-0141-3. PMC7254499 31217577

[3] E. El Agha and V. J. Thannickal , “The Lung Mesenchyme in Development, Regeneration, and Fibrosis,” Journal of Clinical Investigation 133, no. 14 (2023): 170498, 10.1172/JCI170498. PMC10348757 37463440

[4] D. M. Alanis , D. R. Chang , H. Akiyama , M. A. Krasnow , and J. Chen , “Two Nested Developmental Waves Demarcate a Compartment Boundary in the Mouse Lung,” Nature Communications 5, no. 1 (2014): 3923, 10.1038/ncomms4923. PMC4115076 24879355

[5] E. L. Rawlins , C. P. Clark , Y. Xue , and B. L. M. Hogan , “The Id2+ Distal Tip Lung Epithelium Contains Individual Multipotent Embryonic Progenitor Cells,” Development 136, no. 22 (2009): 3741–3745, 10.1242/dev.037317. PMC2766341 19855016

[6] B. E. Rockich , S. M. Hrycaj , H. P. Shih , et al., “Sox9 Plays Multiple Roles in the Lung Epithelium during Branching Morphogenesis,” Proceedings of the National Academy of Sciences of the United States of America 110, no. 47 (2013): E4456–E4464, 10.1073/pnas.1311847110. PMC3839746 24191021

[7] D. R. Chang , D. Martinez Alanis , R. K. Miller , et al., “Lung Epithelial Branching Program Antagonizes Alveolar Differentiation,” Proceedings of the National Academy of Sciences 110, no. 45 (2013): 18042–18051, 10.1073/pnas.1311760110. PMC3831485 24058167

[8] M. E. Kumar , P. E. Bogard , F. Hernán Espinoza , D. B. Menke , D. M. Kingsley , and M. A. Krasnow , “Defining a Mesenchymal Progenitor Niche at Single‐Cell Resolution,” Science 346, no. 6211 (2014): 1258810, 10.1126/science.1258810. PMC4269943 25395543

[9] J. A. Zepp , W. J. Zacharias , D. B. Frank , et al., “Distinct Mesenchymal Lineages and Niches Promote Epithelial Self‐Renewal and Myofibrogenesis in the Lung,” Cell 170, no. 6 (2017): 1134–1148.e10, 10.1016/j.cell.2017.07.034. PMC5718193 28886382

[10] J.‐H. Lee , T. Tammela , M. Hofree , et al., “Anatomically and Functionally Distinct Lung Mesenchymal Populations Marked by Lgr5 and Lgr6,” Cell 170, no. 6 (2017): 1149–1163.e12, 10.1016/j.cell.2017.07.028. PMC5607351 28886383

[11] M. J. Alexander , G. R. Scott Budinger , and P. A. Reyfman , “Breathing Fresh Air into Respiratory Research with Single‐Cell RNA Sequencing,” European Respiratory Review 29, no. 156 (2020): 1–14, 10.1183/16000617.0060-2020. PMC7719403 32620586

[12] S. Bhattacharya , J. A. Myers , C. Baker , et al., “Single‐Cell Transcriptomic Profiling Identifies Molecular Phenotypes of Newborn Human Lung Cells,” Genes 15, no. 3 (2024): 298, 10.3390/genes15030298. PMC10970229 38540357

[13] Z. Xu , X. Wang , L. Fan , et al., “Integrative Analysis of Spatial Transcriptome with Single‐Cell Transcriptome and Single‐Cell Epigenome in Mouse Lungs after Immunization,” iScience 25, no. 9 (2022): 104900, 10.1016/j.isci.2022.104900. PMC9418911 36039299

[14] P. He , K. Lim , D. Sun , et al., “A Human Fetal Lung Cell Atlas Uncovers Proximal‐Distal Gradients of Differentiation and Key Regulators of Epithelial Fates,” Cell 185, no. 25 (2022): 4841–4860, 10.1016/j.cell.2022.11.005. PMC7618435 36493756

[15] P. L. Ståhl , F. Salmén , S. Vickovic , et al., “Visualization and Analysis of Gene Expression in Tissue Sections by Spatial Transcriptomics,” Science 353, no. 6294 (2016): 78–82, 10.1126/science.aaf2403. 27365449

[16] A. Sountoulidis , S. M. Salas , E. Braun , et al., “A Topographic Atlas Defines Developmental Origins of Cell Heterogeneity in the Human Embryonic Lung,” Nature Cell Biology 25, no. 2 (2023): 351–365, 10.1038/s41556-022-01064-x. PMC9928586 36646791

[17] M. F. de Oliveira , J. P. Romero , and M. Chung , “High‐Definition Spatial Transcriptomic Profiling of Immune Cell Populations in Colorectal Cancer,” Nature Genetics 57, no. 6 (2025): 1512–1523, 10.1038/s41588-025-02193-3. PMC12165841 40473992

[18] R. R. Stickels , E. Murray , P. Kumar , et al., “Highly Sensitive Spatial Transcriptomics at Near‐Cellular Resolution with Slide‐SeqV2,” Nature Biotechnology 39, no. 3 (2021): 313–319, 10.1038/s41587-020-0739-1. PMC8606189 33288904

[19] C.‐S. Cho , J. Xi , Y. Si , et al., “Microscopic Examination of Spatial Transcriptome Using Seq‐Scope,” Cell 184, no. 13 (2021): 3559–3572.e22, 10.1016/j.cell.2021.05.010. PMC8238917 34115981

[20] A. Chen , S. Liao , M. Cheng , et al., “Spatiotemporal Transcriptomic Atlas of Mouse Organogenesis Using DNA Nanoball‐Patterned Arrays,” Cell 185, no. 10 (2022): 1777–1792.e21, 10.1016/j.cell.2022.04.003. 35512705

[21] M. Schott , D. León‐Periñán , E. Splendiani , et al., “Open‐ST: High‐Resolution Spatial Transcriptomics in 3D,” Cell 187, no. 15 (2024): 3953–3972.e26, 10.1016/j.cell.2024.05.055. 38917789

[22] Y. You , Y. Fu , L. Li , et al., “Systematic Comparison of Sequencing‐Based Spatial Transcriptomic Methods,” Nature Methods 21, no. 9 (2024): 1743–1754, 10.1038/s41592-024-02325-3. PMC11399101 38965443

[23] Y. Liu , M. Yang , Y. Deng , et al., “High‐Spatial‐Resolution Multi‐Omics Sequencing via Deterministic Barcoding in Tissue,” Cell 183, no. 6 (2020): 1665–1681.e18, 10.1016/j.cell.2020.10.026. PMC7736559 33188776

[24] J. Tollet , A. W. Everett , and M. P. Sparrow , “Spatial and Temporal Distribution of Nerves, Ganglia, and Smooth Muscle during the Early Pseudoglandular Stage of Fetal Mouse Lung Development,” Developmental Dynamics 221, no. 1 (2001): 48–60, 10.1002/dvdy.1124. 11357193

[25] L. J. Freem , S. Escot , D. Tannahill , N. R. Druckenbrod , N. Thapar , and A. J. Burns , “The Intrinsic Innervation Of The Lung Is Derived From Neural Crest Cells As Shown By Optical Projection Tomography in Wnt1‐Cre;YFP Reporter Mice,” Journal of Anatomy 217, no. 6 (2010): 651–664, 10.1111/j.1469-7580.2010.01295.x. PMC3039178 20840354

[26] T. K. Hughes , M. H. Wadsworth , T. M. Gierahn , et al., “Second‐Strand Synthesis‐Based Massively Parallel ScRNA‐Seq Reveals Cellular States and Molecular Features of Human Inflammatory Skin Pathologies,” Immunity 53, no. 4 (2020): 878–894.e7, 10.1016/j.immuni.2020.09.015. PMC7562821 33053333

[27] B. K. Martin , C. Qiu , E. Nichols , et al., “Optimized Single‐Nucleus Transcriptional Profiling by Combinatorial Indexing,” Nature Protocols 18, no. 1 (2023): 188–207, 10.1038/s41596-022-00752-0. PMC9839601 36261634

[28] J. H. Lee , E. R. Daugharthy , J. Scheiman , et al., “Highly Multiplexed Subcellular RNA Sequencing in Situ,” Science 343, no. 6177 (2014): 1360–1363, 10.1126/science.1250212. PMC4140943 24578530

[29] X. Wang , W. E. Allen , M. A. Wright , et al., “Three‐Dimensional Intact‐Tissue Sequencing of Single‐Cell Transcriptional States,” Science 361, no. 6400 (2018): eaat5691, 10.1126/science.aat5691. PMC6339868 29930089

[30] R. Deng , K. Zhang , Y. Sun , X. Ren , and J. Li , “Highly Specific Imaging of MRNA in Single Cells by Target RNA‐Initiated Rolling Circle Amplification,” Chemical Science 8, no. 5 (2017): 3668–3675, 10.1039/c7sc00292k. PMC5437493 28580104

[31] D. Zhang , Y. Deng , P. Kukanja , et al., “Spatial epigenome–transcriptome co‐profiling of mammalian tissues,” Nature 616, no. 7955 (2023): 113–122, 10.1038/s41586-023-05795-1. PMC10076218 36922587

[32] J. Wirth , N. Huber , K. Yin , et al., “Spatial Transcriptomics Using Multiplexed Deterministic Barcoding in Tissue,” Nature Communications 14, no. 1 (2023): 1523, 10.1038/s41467-023-37111-w. PMC10024691 36934108

[33] A. J. C. Russell , J. A. Weir , N. M. Nadaf , et al., “Slide‐Tags Enables Single‐Nucleus Barcoding for Multimodal Spatial Genomics,” Nature 625, no. 7993 (2024): 101–109, 10.1038/s41586-023-06837-4. PMC10764288 38093010

[34] K. Goodwin , B. Lemma , P. Zhang , A. Boukind , and C. M. Nelson , “Plasticity in Airway Smooth Muscle Differentiation during Mouse Lung Development,” Developmental Cell 58, no. 5 (2023): 338–347.e4, 10.1016/j.devcel.2023.02.002. PMC10149112 36868232

[35] K. Goodwin , J. M. Jaslove , H. Tao , M. Zhu , S. Hopyan , and C. M. Nelson , “Patterning the Embryonic Pulmonary Mesenchyme,” iScience 25, no. 3 (2022): 103838, 10.1016/j.isci.2022.103838. PMC8889149 35252804

[36] J. A. Zepp , M. P. Morley , C. Loebel , et al., “Genomic, Epigenomic, and Biophysical Cues Controlling the Emergence of the Lung Alveolus,” Science 371, no. 6534 (2021): abc3172, 10.1126/science.abc3172. PMC8320017 33707239

[37] S. Jiang , Z. Huang , Y. Li , et al., “Single‐Cell Chromatin Accessibility and Transcriptome Atlas of Mouse Embryos,” Cell Reports 42, no. 3 (2023): 112210, 10.1016/j.celrep.2023.112210. 36881507

[38] V. D. Blondel , J. L. Guillaume , R. Lambiotte , and E. Lefebvre , “Fast Unfolding of Communities in Large Networks,” Journal of Statistical Mechanics: Theory and Experiment 2008, no. 10 (2008): P10008, 10.1088/1742-5468/2008/10/P10008.

[39] Y. Ma and X. Zhou , “Spatially Informed Cell‐Type Deconvolution for Spatial Transcriptomics,” Nature Biotechnology 40, no. 9 (2022): 1349–1359, 10.1038/s41587-022-01273-7. PMC9464662 35501392

[40] A. Rao , D. Barkley , G. S. França , and I. Yanai , “Exploring Tissue Architecture Using Spatial Transcriptomics,” Nature 596, no. 7871 (2021): 211–220, 10.1038/s41586-021-03634-9. PMC8475179 34381231

[41] S. V. Paramore , K. Goodwin , and C. M. Nelson , “How to Build an Epithelial Tree,” Physical Biology 19, no. 6 (2022): 061002, 10.1088/1478-3975/ac9e38. 36317265

[42] S. Danopoulos , I. Alonso , M. E. Thornton , et al., “Human Lung Branching Morphogenesis Is Orchestrated by the Spatiotemporal Distribution of ACTA2, SOX2, and SOX9,” American Journal of Physiology‐Lung Cellular and Molecular Physiology 314, no. 1 (2018): L144–L149, 10.1152/ajplung.00379. PMC8312513 28971977

[43] A. K. T. Perl , R. Kist , Z. Shan , G. Scherer , and J. A. Whitsett , “Normal Lung Development and Function after Sox9 Inactivation in the Respiratory Epithelium,” Genesis (United States) 41, no. 1 (2005): 23–32, 10.1002/gene.20093. 15645446

[44] A. Sountoulidis , J. Theelke , A. Liontos , et al., “FGFR Signaling Establishes Spatial Gradients of Secretory Cell Identities along the Airway Proximal‐Distal Axis,” Nature Communications 17, no. 1 (2026): 2651, 10.1038/s41467-026-70842-0. PMC13004988 41851103

[45] M. C. Eblaghie , M. Reedy , T. Oliver , Y. Mishina , and B. L. M. Hogan , “Evidence That Autocrine Signaling through Bmpr1a Regulates the Proliferation, Survival and Morphogenetic Behavior of Distal Lung Epithelial Cells,” Developmental Biology 291, no. 1 (2006): 67–82, 10.1016/j.ydbio.2005.12.006. 16414041

[46] T. Lu , X. Lin , Y. H. Pan , et al., “ADAMTS18 Deficiency Leads to Pulmonary Hypoplasia and Bronchial Microfibril Accumulation,” iScience 23, no. 9 (2020): 101472, 10.1016/j.isci.2020.101472. PMC7476315 32882513

[47] J. C. Herriges , J. M. Verheyden , Z. Zhang , et al., “FGF‐Regulated ETV Transcription Factors Control FGF‐SHH Feedback Loop in Lung Branching,” Developmental Cell 35, no. 3 (2015): 322–332, 10.1016/j.devcel.2015.10.006. PMC4763945 26555052

[48] M. Donadon and M. M. Santoro , “The Origin and Mechanisms of Smooth Muscle Cell Development in Vertebrates,” Development 148, no. 7 (2021): dev197384, 10.1242/dev.197384. 33789914

[49] A. Moiseenko , V. Kheirollahi , C.‐M. Chao , et al., “Origin and Characterization of Alpha Smooth Muscle Actin‐Positive Cells during Murine Lung Development,” Stem Cells 35, no. 6 (2017): 1566–1578, 10.1002/stem.2615. 28370670

[50] K. Goodwin , S. Mao , T. Guyomar , et al., “Smooth Muscle Differentiation Shapes Domain Branches during Mouse Lung Development,” Development 146, no. 22 (2019): dev181172, 10.1242/dev.181172. PMC6899029 31645357

[51] H. Y. Kim , M.‐F. Pang , V. D. Varner , et al., “Localized Smooth Muscle Differentiation Is Essential for Epithelial Bifurcation during Branching Morphogenesis of the Mammalian Lung,” Developmental Cell 34, no. 6 (2015): 719–726, 10.1016/j.devcel.2015.08.012. PMC4589145 26387457

[52] T. Peng , Y. Tian , C. J. Boogerd , et al., “Coordination of Heart and Lung Co‐Development by a Multipotent Cardiopulmonary Progenitor,” Nature 500, no. 7464 (2013): 589–592, 10.1038/nature12358. PMC3758448 23873040

[53] A. M. Goss , Y. Tian , T. Tsukiyama , et al., “Wnt2/2b and β‐Catenin Signaling Are Necessary and Sufficient to Specify Lung Progenitors in the Foregut,” Developmental Cell 17, no. 2 (2009): 290–298, 10.1016/j.devcel.2009.06.005. PMC2763331 19686689

[54] S. Jin , M. V. Plikus , and Q. Nie , “CellChat for Systematic Analysis Of Cell–Cell Communication From Single‐Cell Transcriptomics,” Nature Protocols 20, no. 1 (2024): 180–219, 10.1038/s41596-024-01045-4. 39289562

[55] S. Jin , C. F. Guerrero‐Juarez , L. Zhang , et al., “Inference and Analysis of Cell‐Cell Communication Using CellChat,” Nature Communications 12, no. 1 (2021): 1088, 10.1038/s41467-021-21246-9. PMC7889871 33597522

[56] N. Maeda and M. Noda , “Involvement of Receptor‐like Protein Tyrosine Phosphatase ζ/RPTPβ and Its Ligand Pleiotrophin/Heparin‐binding Growth‐associated Molecule (HB‐GAM) in Neuronal Migration,” Journal of Cell Biology 142, no. 1 (1998): 203–216, 10.1083/jcb.142.1.203. PMC2133018 9660874

[57] H. Rauvala , “An 18‐kd Heparin‐Binding Protein Of Developing Brain That Is Distinct From Fibroblast Growth Factors,” EMBO Journal 8, no. 10 (1989): 2933–2941, 10.1002/j.1460-2075.1989.tb08443.x. PMC401361 2583087

[58] R. M. Gilbert , L. E. Schappell , and J. P. Gleghorn , “Defective Mesothelium and Limited Physical Space Are Drivers of Dysregulated Lung Development in a Genetic Model of Congenital Diaphragmatic Hernia,” Development (Cambridge) 148 (2021): dev199460, 10.1242/DEV.199460. PMC8180258 34015093

[59] T. H. Lüdtke , C. Rudat , J. Kurz , et al., “Mesothelial Mobilization in the Developing Lung and Heart Differs in Timing, Quantity, and Pathway Dependency,” American Journal of Physiology. Lung Cellular and Molecular Physiology 316 (2019): 767–783, 10.1152/ajplung.00212.2018.-The. 30702346

[60] J. Que , B. Wilm , H. Hasegawa , F. Wang , D. Bader , and B. L. M. Hogan , “Mesothelium Contributes to Vascular Smooth Muscle and Mesenchyme during Lung Development,” Proceedings of the National Academy of Sciences 105, no. 43: 16626–16630. 10.1073/pnas.0808649105 PMC2567908 18922767

[61] L. Aven and X. Ai , “Mechanisms of Respiratory Innervation during Embryonic Development,” Organogenesis 9, no. 3 (2013): 194–198, 10.4161/org.24842. PMC3896590 23974176

[62] R. Azzoni , O. Perdijk , N. L. Harris , and B. J. Marsland , “Neuroimmunology of the Lung,” Annual Review of Immunology 42, no. 1 (2024): 57–81, 10.1146/annurev-immunol-083122. 37989144

[63] A. Langsdorf , K. Radzikinas , A. Kroten , S. Jain , and X. Ai , “Neural Crest Cell Origin and Signals for Intrinsic Neurogenesis in the Mammalian Respiratory Tract,” American Journal of Respiratory Cell and Molecular Biology 44, no. 3 (2011): 293–301, 10.1165/rcmb.2009-0462OC. PMC3159091 20139349

[64] M. Guo , M. P. Morley , C. Jiang , et al., “Guided Construction of Single Cell Reference for Human and Mouse Lung,” Nature Communications 14, no. 1 (2023): 4566, 10.1038/s41467-023-40173-5. PMC10387117 37516747

[65] Y. Yin , J. R. Koenitzer , D. Patra , et al., “Identification of a Myofibroblast Differentiation Program during Neonatal Lung Development,” Development 151, no. 9 (2024): dev202659, 10.1242/dev.202659. PMC11165721 38602479

[66] M. Riccetti , J. J. Gokey , B. Aronow , and A. K. T. Perl , “The Elephant in the Lung: Integrating Lineage‐Tracing, Molecular Markers, and Single Cell Sequencing Data to Identify Distinct Fibroblast Populations during Lung Development and Regeneration,” Matrix Biology 91‐92 (2020): 51–74, 10.1016/j.matbio.2020.05.002. PMC7434667 32442602

[67] M. Chen , J. Lv , Q. Zhang , et al., “Spatial Transcriptomics Unveils the Blueprint of Mammalian Lung Development,” Genomics, Proteomics & Bioinformatics (2026): qzaf053, 10.1093/gpbjnl/qzaf053/8160015. PMC13466558 40493888

[68] J. M. Jaslove , K. Goodwin , A. Sundarakrishnan , et al., “Transmural Pressure Signals through Retinoic Acid to Regulate Lung Branching,” Development 149, no. 2 (2022): dev199726, 10.1242/dev.199726. PMC8917413 35051272

[69] J. F. Navarro , J. Sjöstrand , F. Salmén , J. Lundeberg , and P. L. Ståhl , “ST Pipeline: An Automated Pipeline for Spatial Mapping of Unique Transcripts,” Bioinformatics 33, no. 16 (2017): 2591–2593, 10.1093/bioinformatics/btx211. 28398467

[70] T. R. Sztanka‐Toth , M. Jens , N. Karaiskos , and N. Rajewsky , “Spacemake: Processing and Analysis of Large‐Scale Spatial Transcriptomics Data,” GigaScience 11 (2022): giac064, 10.1093/gigascience/giac064. PMC9295369 35852420

[71] S. Choudhary and R. Satija , “Comparison and Evaluation of Statistical Error Models for ScRNA‐Seq,” Genome Biology 23, no. 1 (2022): 27, 10.1186/s13059-021-02584-9. PMC8764781 35042561

[72] G. Palla , H. Spitzer , M. Klein , et al., “Squidpy: A Scalable Framework for Spatial Omics Analysis,” Nature Methods 19, no. 2 (2022): 171–178, 10.1038/s41592-021-01358-2. PMC8828470 35102346

