# Supplementary material for: Mapping Embryonic Mouse Lung Development Using Enhanced Spatial Transcriptomics
Source: Adv Sci (Weinh). 2026 Sep 27:e77678. Online ahead of print. doi: 10.1002/advs.77678 (PMC13616284; doi:10.1002/advs.77678)
Supplement: Supplementary file 1 [file ADVS-9999-e77678-s002.docx]

Supporting Information

**Mapping embryonic mouse lung development using enhanced spatial transcriptomics**

Pengfei Zhang, Benjamin K. Law, Niles Huang, Katharine Goodwin, Celeste M. Nelson^*^, and Michelle M. Chan^*^

**Supplementary Table 1.** DNA sequences of the template-switch oligo, ligation linker, and barcode A and B oligonucleotides used in Enhanced_DBiT_v1.

**Supplementary Table 2.** DNA sequences of the reverse transcription primer, ligation linkers, and barcode A and B oligonucleotides used in Enhanced_DBiT_v2.

**Supplementary Table 3.** DNA sequences of the random priming primer for second-strand synthesis, cDNA amplification primer, and sequencing index oligonucleotides.

**Figure. S1. Optimized DBiT-seq workflow (Enhanced_DBiT_v1) – related to Figure. 1.** Schematic of steps optimized in a previously published DBiT-seq workflow to enable cellular resolution spatial transcriptomics with high sensitivity.

**Figure. S2. Optimized DBiT-seq workflow (Enhanced_DBiT_v1) shows superior cDNA and UMI recovery - related to Figure. 1.** (**a**) Schematic showing comparison between two fixation conditions: PFA with DEPC and PFA without DEPC. (**b**) Graph of cDNA fragment length as a function of fixation condition. (**c**) Schematic showing comparison between two other fixation conditions: PFA with DEPC and methanol/DSP with DEPC. (**d**) Graph of cDNA fragment length as a function of fixation using PFA versus methanol/DSP. (**e**) Schematic showing second-strand synthesis via random priming. (**f**) Graph of cDNA fragment length with or without second-strand synthesis via random priming.

**Figure. S3. Optimized DBiT-seq workflow (Enhanced_DBiT_v2) – related to Figure. 1.** (**a**) Schematic of steps of Enhanced_DBiT_v2 using bulk RT. (**b**) Saturation curves of different spatial transcriptomic mapping technologies.

**Figure. S4. Embryonic lung replicates and integration of spatial transcriptomic data with scRNA-seq – related to Figure. 2.** (**a**) Brightfield images of replicates at each stage of development. Scale bars, 200 µm. (**b**) UMAP of integration of spatial transcriptomic data with reference scRNA-seq data. (**c**) Composition of clusters at each stage of development.

**Figure. S5**. **Spatial transcriptomic data quality control and plots of feature gene markers – related to Figure. 2.** (**a-b**) Violin plots of UMI/pixel and gene/pixel across the different replicates of spatial transcriptomic mapping. (**c**) UMAPs of marker genes for the different cell types in the lung, including *Epcam* and *Wnt7b* (Epithelium), *Sox9* (Precartilage and Distal Epithelium), *Sox2* (Proximal Epithelium), *Wnt2* (Mesenchymal Progenitors), *Wt1* and *Aldh1a2* (Mesothelium), *Pdgfrb* (Pericytes), *Prdm16* (Precartilage), *Ptch1*, *Mylk*, and *Actg2* (Smooth Muscle), *Cdh5* (Vascular Endothelium), *Sox10* (Neurons), *Tnni1* (Heart), and *Fcer1g* (Immune Cells).

**Figure. S6**. **UMAPs split by timepoints and each individual sample – related to Figure. 2**. (**a**) UMAPs spit by developmental timepoints. (**b**) UMAPs split by each individual spatial sample. (**c**) UMAPs split by each individual single-cell RNA-seq (scRNA-seq) sample.

**Figure. S7. Spatial mapping of major cell types across different stages of development – related to Figure. 2.** (**a-c**) Color-coded maps of cells from each cluster, as defined in Figure. 2b, in each replicate at each stage examined.

**Figure. S8. Spatial mapping of Heart and Blood cells across different stages of development - related to Figure. 2.** (**a**) Color-coded maps of Heart cells in each replicate at each stage examined. (**b**) Color-coded maps of Blood cells together with Vascular Endothelium, in each replicate at each stage examined.

**Figure. S9. Cell type deconvolution analysis – related to Figure. 2**. (**a**) Color-coded map of cell identities (right), as inferred by cell deconvolution via CARD using scRNA-seq data (left) as a reference. (**b**) Color-coded maps of epithelium, smooth muscle, and pre-cartilage, as predicted by the deconvoluted data, versus spatial mapping based on annotation of integrated spatial and scRNA-seq data. (**c**) Color-coded spatial pie-chart plots of cell identities for all replicates, as inferred from cell type deconvolution.

**Figure. S10. Filtering spatial pixels based on cell type deconvolution analysis– related to Figure. 2**. (**a**) Histogram of maximum predicted cell-type proportion, as inferred by cell deconvolution via CARD using scRNA-seq data. A threshold of 0.5 was applied to filter pixels lacking a dominant cell type. (**b**) UMAP of spatial pixels passing filtration. (**c**) Heatmap of pairwise gene expression correlations between cell types in the scRNA-seq reference, with hierarchical clustering (dendrogram) shown at left.

**Figure. S11. Spatial mapping of proximal and distal epithelial cells across different stages of development - related to Figure. 3.** (**a-c**) Color-coded maps of proximal and distal epithelium, in each replicate at each stage examined.

**Figure. S12. Spatial maps of epithelial markers at each stage of lung development – related to Figure. 3.** Color-coded maps of (**a-c**) *Sox9* and (**d-f**) *Id2* in each replicate at each stage examined. For ease of visualization, epithelial cells are highlighted with larger, darker pixels; spatial resolution is unchanged.

**Figure. S13. Spatial maps of epithelial markers at each stage of lung development – related to Figure. 3.** Color-coded maps of (**a-c**) *Etv5* in each replicate at each stage examined. For ease of visualization, epithelial cells are highlighted with larger, darker pixels; spatial resolution is unchanged.

**Figure. S14. Fluorescence in situ hybridization for proximal and distal epithelial markers – related to Figure. 3.** (**a-c**) Top: fluorescence in situ hybridization for *Shh*, *Sox2*, *Sox9*, counterstained with DAPI at each stage examined. Bottom: zoomed-in view of proximal regions boxed in top. Arrows point to the pixels with high *Sox9* signal. Scale bars, 100 µm.

**Figure. S15. Analysis of *Sox2* and *Sox9* expression in the epithelium – related to Figure. 3.** (**a**) Scatter plots of *Sox2* and *Sox9* intensity, from fluorescence in situ hybridization analysis. (**b**) Graph showing quantification of *Sox2*^+^*Sox9*^+^ double-positive epithelial cells in scRNA-seq reference data. (**c**) Immunofluorescence staining of *E*11.5 lungs showing SOX2 and SOX9. Scale bars, 100 µm (top row) and 50 µm (bottom row).

**Figure. S16. Spatial mapping of proximal and distal epithelial cells across different stages of development – related to Figure. 3.** (**a-c**) Color-coded maps of epithelial subclusters, as defined in Figure. 2d, in each replicate at each stage examined. (**d**) Violin plots of additional proximal (*Igfbp5* and *Ccnd2*) and distal (*Id2* and *Sftpc*) markers in each epithelial subcluster in scRNA-seq data.

**Figure. S17. Gene markers of epithelial subclusters and pseudotime inference of epithelial differentiation trajectory – related to Figure. 3.** (**a-b**) Volcano plots for differential gene expression between Proximal Intermediate and other epithelial cells, and between merged intermediate and other epithelial cells in scRNA-seq data. (**c**) Violin plots of markers for intermediate (*Smoc2*), Proximal Epithelial (*Adamtsl1*), and Distal Epithelial cells (*Adamts18*, *Etv4*, and *Thbd*) in scRNA-seq data. (**d**) Violin plots of *Fgfbp1* and *Icam1* in scRNA-seq data. (**e**) UMAP of epithelial pseudotime inference. (**f**) Graphs of density of cell states along the pseudotime from (e). (**g**) Color-coded maps of spatial pseudotime at each stage of lung development. (**h**) Heatmap of gene markers along the pseudotime trajectory from (e).

**Figure. S18. Fluorescence in situ hybridization of *Shh*, *Smoc2*, and *Cldn10* – related to Figure. 3.** (**a-c**) Fluorescence images of hybridization analysis for DAPI, *Shh*, *Smoc2*, and *Cldn10* at each stage. Bottom rows are zoomed-in views of insets indicated by boxes, showing DAPI, *Shh*, *Smoc2*, and *Cldn10*. Scale bars, 200 µm for the upper row, 50 µm for the bottom row at each stage.

**Figure. S19. Spatial maps of markers for Distal Epithelium – related to Figure. 3.** (**a-c**) Spatial maps of Distal Epithelium-enriched genes *Bmp4*, *Adamts18*, and *Thbd* at each stage examined.

**Figure. S20. Gene markers of mesenchymal subclusters – related to Figure. 4.** (**a**) Heatmap of gene markers for mesenchymal subclusters using scRNA-seq data. (**b**) Color-coded maps of Mesenchymal Progenitor 1 (MP1), Mesenchymal Progenitor 2 (MP2), Mesenchymal Progenitor 3 (MP3), Smooth Muscle Progenitor (SMP), Pericytes, and Precartilage at each stage examined. (**c**) UMAP of pseudotime inference for the smooth-muscle trajectory. (**d**) Spatial pseudotime mapping of smooth-muscle trajectory at each stage of lung development.

**Figure. S21. Spatial maps of Matrix Mesenchyme and its gene markers – related to Figure. 4.** (**a**) Spatial maps of Matrix Mesenchyme’s top markers *Col1a1* and *Col1a2*. (**b**) Violin plots of *Osr1* and *Sox9* in mesenchymal populations, including Mesenchymal Progenitor 1-3 (MP1-3), Pericytes, Smooth Muscle Progenitors (SMP), Smooth Muscle Cells (SMC), Matrix Mesenchyme (MatrixM), and Precartilage in scRNA-seq data.

**Figure. S22. Fluorescence in situ hybridization for *Prrx1*, *Osr1*, and *Sox9* – related to Figure. 4.** (**a-c**) Fluorescence images of hybridization analysis for DAPI, *Prrx1*, *Osr1*, and *Sox9* at each stage. Bottom rows show zoomed-in views of the boxed regions from the corresponding upper-row images. Scale bars, 200 µm for the upper row, 50 µm for the bottom row at each stage. The raw images were rotated and cropped to orient the trachea upward, and the empty space was filled with a rectangular black box.

**Figure. S23. Fluorescence in situ hybridization for *Prrx1*, *Col1a1*, and *Ptn* – related to Figure. 4.** Fluorescence images of hybridization analysis for DAPI, *Prrx1*, *Col1a1*, and *Ptn* at *E*13.5. Bottom rows show zoomed-in views of the boxed regions from the corresponding upper-row images. Scale bars, 200 µm for the upper row, 50 µm for the bottom rows.

**Figure. S24. Expression of Ptn receptors and spatial localization of Matrix Mesenchyme and Neurons – related to Figure. 4.** (**a-c**) Color-coded maps of Matrix Mesenchyme and Neurons in each replicate at each stage examined. (**d**) Violin plot of *Ptn.* (**e**) Spatial maps of *Ptn* at *E*12.5. (**f**) Dot plot of gene expression for Ptn receptors *Sdc3* and *Ptprz1* in scRNA-seq data.

 **Figure. S25. Contribution of putative esophageal tissue to Matrix Mesenchyme and its proximity to neurons – related to Figure. 4.** (**a**) Color-coded maps of putative esophageal and lung tissue in each replicate at each stage examined. (**b**) UMAP of putative esophageal tissue. (**c**) Cell type composition in the putative esophageal tissue. (**d**) Z-score plot for neighborhood-enrichment analysis of Neurons after excluding putative esophageal pixels. MatrixM: Matrix Mesenchyme; VasEndo: Vascular Endothelium; SMP: Smooth Muscle Progenitor; ProxEpithelium: Proximal Epithelium; MP: Mesenchymal Progenitors.

**Figure. S26. Spatial localization of PTN in the embryonic lung – related to Figure. 4.** (**a**) Immunofluorescence staining for TuJ1 (neuronal marker), PTN, and ECAD (epithelial marker) in whole lung section. Scale bars, 100 µm. (**i-iii**) Zoomed-in images of TuJ1, PTN, and ECAD at three different locations. Scale bars, 50 µm. (**b**) Negative control of immunofluorescence staining for PTN while TuJ1 and ECAD were stained as usual. Scale bars, 100 µm.

**Figure. S27. Spatial localization of PTN^+^ cells in the embryonic lung – related to Figure. 4.** (**a-c**) Immunofluorescence staining for WT1 (mesothelial marker), TuJ1 (neuronal marker), PTN, and DAPI. Bottom rows are zoomed-in views of insets indicated by boxes, showing WT1, PTN, and DAPI. Scale bars, 200 µm for the upper rows, 50 µm for the bottom rows.

**Figure. S28. Lung explant culture using PTN function-blocking antibody – related to Figure. 4.** (**a**) Ex vivo culture of lung explant using anti-PTN functional blocking or control antibody. (**b**) Immunofluorescence analysis for ECAD (green) and TuJ1 (magenta) in lung explants cultured in the presence of anti-PTN or control antibody for 36 h. The filaments identified in Imaris are highlighted in yellow and enclosed within red dotted boxes. Scale bars, 100 µm.

**Figure. S29. Differential gene expression analysis between different branching modes – related to Figure. 2.** (**a-c**) Color-coded maps of proximal and distal epithelium, domain branching, and bifurcation in each replicate we selected. (**d**) Volcano plot of differential gene expression analysis in Mesenchyme Progenitor between different branching modes.

**Figure. S30. UMAPs of Alveolar Fibroblast 2 – related to Figure. 4.** UMAP of scRNA-seq reference dataset for mouse lung (Mouse CellCards Multi-Study CellRef 1.0 Atlas), with Alveolar Fibroblast 2 (AF2) highlighted (bottom center) and feature plots of AF2’s top markers, including *Ptn*, *Prrx1*, *Osr1*, *Col1a1*, *Col1a2*, *Igfbp5*, and *Mfap5*.
